# Direct medical costs of ischemic heart disease in urban Southern China: a 5-year retrospective analysis of an all-payer health claims database in Guangzhou City

**DOI:** 10.3389/fpubh.2023.1146914

**Published:** 2023-05-09

**Authors:** Peixuan Xie, Xuezhu Li, Feifan Guo, Donglan Zhang, Hui Zhang

**Affiliations:** ^1^Department of Health Policy and Management, School of Public Health, Sun Yat-sen University, Guangzhou, China; ^2^Division of Health Services Research, New York University Long Island School of Medicine, Mineola, NY, United States

**Keywords:** ischemic heart disease, direct medical costs, health insurance, time trends, China, out-of-pocket (OOP) expenses

## Abstract

**Introduction:**

This study aimed to estimate the direct medical costs and out-of-pocket (OOP) expenses associated with inpatient and outpatient care for IHD, based on types of health insurance. Additionally, we sought to identify time trends and factors associated with these costs using an all-payer health claims database among urban patients with IHD in Guangzhou City, Southern China.

**Methods:**

Data were collected from the Urban Employee-based Basic Medical Insurance (UEBMI) and the Urban Resident-based Basic Medical Insurance (URBMI) administrative claims databases in Guangzhou City from 2008 to 2012. Direct medical costs were estimated in the entire sample and by types of insurance separately. Extended Estimating Equations models were employed to identify the potential factors associated with the direct medical costs including inpatient and outpatient care and OOP expenses.

**Results:**

The total sample included 58,357 patients with IHD. The average direct medical costs per patient were Chinese Yuan (CNY) 27,136.4 [US dollar (USD) 4,298.8] in 2012. The treatment and surgery fees were the largest contributor to direct medical costs (52.0%). The average direct medical costs of IHD patients insured by UEBMI were significantly higher than those insured by the URBMI [CNY 27,749.0 (USD 4,395.9) vs. CNY 21,057.7(USD 3,335.9), *P* < 0.05]. The direct medical costs and OOP expenses for all patients increased from 2008 to 2009, and then decreased during the period of 2009–2012. The time trends of direct medical costs between the UEBMI and URBMI patients were different during the period of 2008-2012. The regression analysis indicated that the UEBMI enrollees had higher direct medical costs (*P* < 0.001) but had lower OOP expenses (*P* < 0.001) than the URBMI enrollees. Male patients, patients having percutaneous coronary intervention operation and intensive care unit admission, patients treated in secondary hospitals and tertiary hospitals, patients with the LOS of 15–30 days, 30 days and longer had significantly higher direct medical costs and OOP expenses (all *P* < 0.001).

**Conclusions:**

The direct medical costs and OOP expenses for patients with IHD in China were found to be high and varied between two medical insurance schemes. The type of insurance was significantly associated with direct medical costs and OOP expenses of IHD.

## Introduction

Ischemic heart disease (IHD) is a major public health concern worldwide, encompassing acute myocardial infarction, chronic stable angina, chronic IHD, and associated heart failure, (1). According to the Global Burden of Disease, Injuries, and Risk Factors Study 2019, there were an estimated 197 million prevalent cases of IHD globally in 2019 (2), making it the leading cause of mortality globally (3). In 2019 alone, IHD was responsible for ~182 million disability-adjusted life years (DALY) and 9.14 million deaths (1). The financial burden of IHD on healthcare expenditures is also significant (4). In the United States (US), the direct and indirect costs of IHD were estimated to be in US dollars (USD) 142.5 billion (5).

In China, the incidence of IHD was ~59.1/100,000 in 2012 (6), and it was predicted that there would be 38.6 million IHD events from 2010 to 2030 (7). The morbidity and mortality of IHD have been continuously increasing (8). In 2015, the total mortality of IHD in China was 22,117/100,000, accounting for 1.51 million deaths (9). In contrast to the decline of IHD mortality in developed countries (3, 10), the standardized mortality rate of IHD has been increasing annually in China, particularly among older adults (9, 11, 12). Population growth and aging have resulted in a steady increase in the burden of IHD (13, 14). It has been estimated that the economic burden of IHD is heaviest among various types of disease in China, accounting for more than Chinese Yuan (CNY) 109.6 billion in 2013 (15).

In order to improve access to healthcare services and provide financial protection for patients, China has launched two main basic health insurance schemes for the urban Chinese population: the Urban Employee-based Basic Medical Insurance (UEBMI) and Urban Resident-based Basic Medical Insurance (URBMI) (16, 17). These two schemes differed considerably in terms of financing, coverage of health services, and reimbursement rates (18). The UEBMI scheme is financed by contributions from both employers and employees (19), while the URBMI scheme is financed by individual premiums and government subsidies (17). UEBMI enrollees have more comprehensive service coverage and higher reimbursement rates than URBMI enrollees (16). It is important to consolidate health insurance schemes and achieve universal health coverage for Chinese people (20). Therefore, estimating the direct medical costs of IHD across different medical insurance schemes provides essential information for policymakers in China.

Several studies have estimated the direct medical costs of IHD in both developed and developing countries (21–27). A study using claims data from the US Medicare population reported that the per-patient expenditures averaged USD 22,921.0 in 2002 (USD 28,288.5 in 2012) (21). In England, a linked cohort study showed that the mean healthcare costs for stable IHD patients in the 1st year were Great Britain Pound (GBP) 3,133.0 in 2012 (USD 4,501.4 in 2012) (22). In Poland, the average annual cost of treating IHD was European Monetary Unit (EUR) 2,254.17 in 2005 (USD 3,046.26 in 2012) (23). The average annual costs for treating IHD in Korea was USD 1,835 in 2005 (USD 2,099 in 2012) (24). A retrospective cohort study in Brazil estimated that the mean annual costs of IHD were USD 1,522.0 in 2015 (USD 1,452.4 in 2012) (25). A prevalence-based study in two major hospitals from Cameroon reported that the average annual direct medial costs of care per IHD patient were USD 2,400.0 in 2017 (USD 2,227.4 in 2012) (26). In Iran, total costs per IHD patient in 1 year were estimated to be Iranian Rial (IRR) 63,452,290.2 in 2017 (USD 1,507.0 in 2012) (27).

Only a few studies have examined the direct medical costs of IHD in China (28–30). Le et al. (30) evaluated the economic burden and costs of IHD in rural Yunnan Province, Southwest China through a cross-sectional survey, and found that the direct medical costs of IHD were estimated to be USD 1,156.2 in 2010 (USD 1,203.0 in 2012). Another study conducted in the rural area of Liaoning province, Northern China with IHD inpatients from discharge records of ten township hospitals discovered that the average hospitalization costs were CNY 6,249.97 (USD 1,012.47) in 2014 (USD 976.60 in 2012) (28). But these two studies only included rural patients, and Le et al.'s study (30) focused on the macroeconomic burden of IHD, while Wang et al.'s study (28) only evaluated the hospitalization costs of IHD in township hospitals where only medication therapy was available. With regards to IHD patients in urban regions, Ding et al. (29) analyzed the direct inpatient costs of IHD in one single tertiary hospital in Xi'an City and reported that the mean hospitalization expenditures were USD 6,791.38 in 2015 (USD 6,481.00 in 2012). However, the samples of Ding et al.'s study (29) were recruited from only one tertiary hospital and might not represent the urban population in China. Furthermore, none of the above China-based studies compared the direct medical costs of IHD including both inpatient and outpatient care under different types of health insurance schemes and did not examine the time trends of medical costs in China.

Different from previous studies, this research aimed to analyze data collected from all different levels of hospitals to estimate the direct medical costs of both inpatient and outpatient care related to IHD by different type of health insurance and figure out the time trends and the factors associated with such direct medical costs and out-of-pocket (OOP) expenses among urban patients with IHD in Guangzhou City, Southern China.

## Method

### Data source

This study collected data from the UEBMI and URBMI claims databases in Guangzhou City between 2008 and 2012, which contained sociodemographic information, medical conditions, hospitalization costs and outpatient costs based on actual amounts paid to all different levels of hospitals. Guangzhou, the capital city of Guangdong province, is one of the largest municipalities in Southern China. Until the year of 2012, there were 93.4% citizens covered by these two urban health insurance schemes in Guangzhou City (31). This study population was a better representation of the Chinese urban population, which represented over 60% of total population in China (32), than that in previous China-based studies.

### Study design

We conducted a retrospective observational study with a cross-sectional design to evaluate the direct medical costs of IHD. This study collected all insurance claims on hospitalization care from January 1, 2008, to December 31, 2012 with the primary diagnosis of IHD according to the International Classification of Disease Tenth Revision (ICD-10, disease codes: I20-I25) (33). Besides, outpatient records of the same period were merged from the outpatient claims database using unique patient identification code. This study excluded patients aged <18 years old. Finally, 58,357 IHD patients were involved in this study, including 53,014 UEBMI enrollees and 5,343 URBMI enrollees.

Direct medical costs referred to the annual medical costs per patient with IHD, including outpatient costs and inpatient costs according to different types of health services. From the payer perspective, the direct medical costs, outpatient and inpatient costs all included individual OOP expenses and the reimbursement amounts. Individual OOP expenses were the costs that were not covered by health insurance and must be paid by patients themselves. In terms of cost composition, direct medical costs consisted of western drug fees, traditional Chinese drug fees, treatment and surgery fees, bed fees, laboratory and diagnostic fees and other fees. The western drug fees were expenses for western medicines while the traditional Chinese drug fees were spending on Chinese patent drugs and Chinese herbal medicines. Treatment and surgery fees were total spending of surgical procedures, medical consumable materials, radiation therapy, oxygen therapy, anesthesia, blood transfusion, and the other forms of treatment excluding medicine therapy. Surgical procedures included catheter-based procedures such as percutaneous coronary intervention (PCI) (34). Bed fees were spending on inpatient accommodation. Laboratory and diagnostic fees were expenses on physical examinations and biochemical test. Other fees were expenses on other services such as air conditioner. All costs were inflated to year 2012 Chinese Yuan (CNY) according to the urban residents Consumer Price Index (CPI) of Guangzhou City (31). The currency exchange rate between US dollar and Chinese Yuan was: USD1.0 = CNY6.3125 in 2012.

The factors associated with total direct medical costs and OOP expenses for IHD patient were selected based on literature and the Andersen's behavioral model of health services use (35). Individual influential factors were selected from the following three sections: (1) predisposing characteristics: factors that induce individuals to health services utilization [such as gender (28), age (25)]; (2) enabling characteristics: factors that foster or hinder health services use [for example, types of insurance (29), hospital levels (36, 37)]; (3) need characteristics: conditions that physicians consider as requiring professional treatment [such as, length of stay (LOS) (22, 26), intensive care unit (ICU) admission (38) and operation of PCI (39, 40), presence of comorbidities (24, 25)].

### Measures and variables

In this study, the dependent variable was annual direct medical expenditures and OOP expenses per patient with IHD, including inpatient and outpatient expenses. The primary independent variable was the type of insurance (patients insured by the scheme of UEBMI or URBMI).

The other covariates included age, gender, ICU admission, operation of PCI, hospital levels, LOS, and presence of comorbidities. Age was categorized into four groups: 18–59 years old, 60–69 years old, 70–79 years old, and 80 years old and above. Gender was dichotomized as female and male. We chose two dummy variables as proxy measures for the severity of disease—whether having an ICU admission, and having the operation of PCI (34). Hospital level was classified into three levels—primary, secondary and tertiary according to different bed size and functional orientation (41). The LOS was divided into three categories: <15 days, 15–29 days, 30 days and longer. Comorbidities were measured as binary variables for the following conditions—whether having a diagnosis of hypertension and diabetes. Years were measured as binary variables for controlling the impact of policy changes across the years.

### Statistical analysis

Descriptive statistics (frequency, percentage, mean, and standard deviation (SD), median and 25–75th percentiles) were used for demographic characteristics and costs. Due to the skewed distribution of direct medical expenditure and OOP expenses, the Kruskal-Wallis Rank Sum test was used to identify the differences in cost composition between the two types of insurance. We also described the time trends of direct medical costs and OOP expenses across different types of insurance. The Friedman's two-way non-parametric analysis of variance test was performed to figure out the disparities in patient characteristics related to direct medical expenditure and OOP expenses by types of insurance. The Extended Estimating Equations (EEE) model (42) was selected to find out the potential associated factors of direct medical expenses and OOP expenses for IHD patients. The EEE model was a flexible parametric link function approach, offering an added degree of flexibility over the standard generalized linear model, while retaining enough model structure to estimate easily (42). This EEE approach has been employed to conduct cost estimation in literature (43–45). Additionally, the heterogeneity analysis was used to examine the differences in the association between types of insurance and direct medical costs as well as OOP expenses among different subgroups. Statistical analysis was conducted *via* R language version 4.1.3 and STATA version 15.0 (STATA Corporation, Collection Station, TX, USA).

## Results

### Patient characteristics

A large sample of 58,357 patients with IHD were in this study. Most of the patients were insured by the UEBMI scheme (90.8%) and the rest were covered by the URBMI scheme (9.2%) (see Table 1). More than half of the patients were female (51.5%). The average age for all sample was 72.0 years old (SD = 11.4). The mean age in the UEBMI subgroup was 71.8 years old (SD = 11.4), while under the URBMI scheme the mean age was 74.1 years old (SD = 11.0). Overall, patients aged 70–79 years old (36.5%) outnumbered the other age groups. The majority of patients (64.9%) received treatment in tertiary hospitals, and 22.9% patients chose secondary hospitals. 16.9% patients underwent PCI operation and 0.1% patients had ICU admission. The mean LOS was 16.1 days (SD = 21.5) and most of patients stayed in the hospitals for <15 days (66.9%). Among the total sample, 23.2% of them had hypertension and 7.2% had diabetes mellitus.

**Table 1:** Characteristics of patients with ischemic heart disease.

**Variables**	**Overall**	**UEBMI**	**URBMI**
No. patients	58,357	53,014	5,343
**Gender**			
Female	30,078 (51.5)	26,608 (50.2)	3,470 (64.9)
Male	28,279 (48.5)	26,406 (49.8)	1,873 (35.1)
**Age (years)**			
Mean ± SD	72.0 ± 11.4	71.8 ± 11.4	74.1 ± 11.0
Median (25th-75th)	74.0 (64.0-80.0)	74.0 (64.0-80.0)	75.0 (67.0-82.0)
**Age group**			
18≤Age<59	9,129 (15.6)	8,563 (16.2)	566 (10.6)
60≤Age<69	12,016 (20.6)	10,912 (20.6)	1,104 (20.7)
70≤Age<79	21,326 (36.5)	19,409 (36.6)	1,917 (35.9)
≥80	15,886 (27.2)	14,130 (26.7)	1,756 (32.9)
**Hospital level**			
Primary	7,100 (12.2)	6,220 (11.7)	880 (16.5)
Secondary	13,381 (22.9)	11,917 (22.5)	1,464 (27.4)
Tertiary	37,876 (64.9)	34,877 (65.8)	2,999 (56.1)
ICU admission	80 (0.1)	73 (0.1)	7 (0.1)
PCI operation	9,846 (16.9)	9,240 (17.4)	606 (11.3)
**Length of stay (days)**			
Mean ± SD	16.1 ± 21.5	16.2 ± 22.0	14.3 ± 16.2
Median (25th-75th)	11.0 (7.0-17.0)	11.0 (7.0-17.0)	10.0 (7.0-16.0)
Days < 15	39,037 (66.9)	35,285 (66.6)	3,752 (70.2)
15≤Days<30	13,274 (22.7)	12,128 (22.9)	1,146 (21.4)
Days ≥ 30	6,046 (10.4)	5,601 (10.6)	445 (8.3)
**Admission year**			
Year 2008	7,832 (13.4)	7,510 (14.2)	322 (6.0)
Year 2009	9,792 (16.8)	8,835 (16.7)	957 (17.9)
Year 2010	9,547 (16.4)	8,671 (16.4)	876 (16.4)
Year 2011	13,945 (23.9)	12,616 (23.8)	1,329 (24.9)
Year 2012	17,241 (29.5)	15,382 (29.0)	1,859 (34.8)
**Comorbidities**			
None	42,024 (72.0)	38,096 (71.9)	3,928 (73.5)
Hypertension	13,544 (23.2)	12,332 (23.3)	1,212 (22.7)
Diabetes mellitus	4,177 (7.2)	3,870 (7.3)	307 (5.7)

*n* (%) for categorical variables and mean ± standard deviation (SD) or median (25–27th) for continuous variables; URBMI, Urban Resident-based Basic Medical Insurance scheme; UEBMI, Urban Employee-based Basic Medical Insurance scheme; ICU, Intensive Care Unit; PCI, Percutaneous Coronary Intervention.

### Direct medical costs and costs composition by insurance type

**Figure 1:**
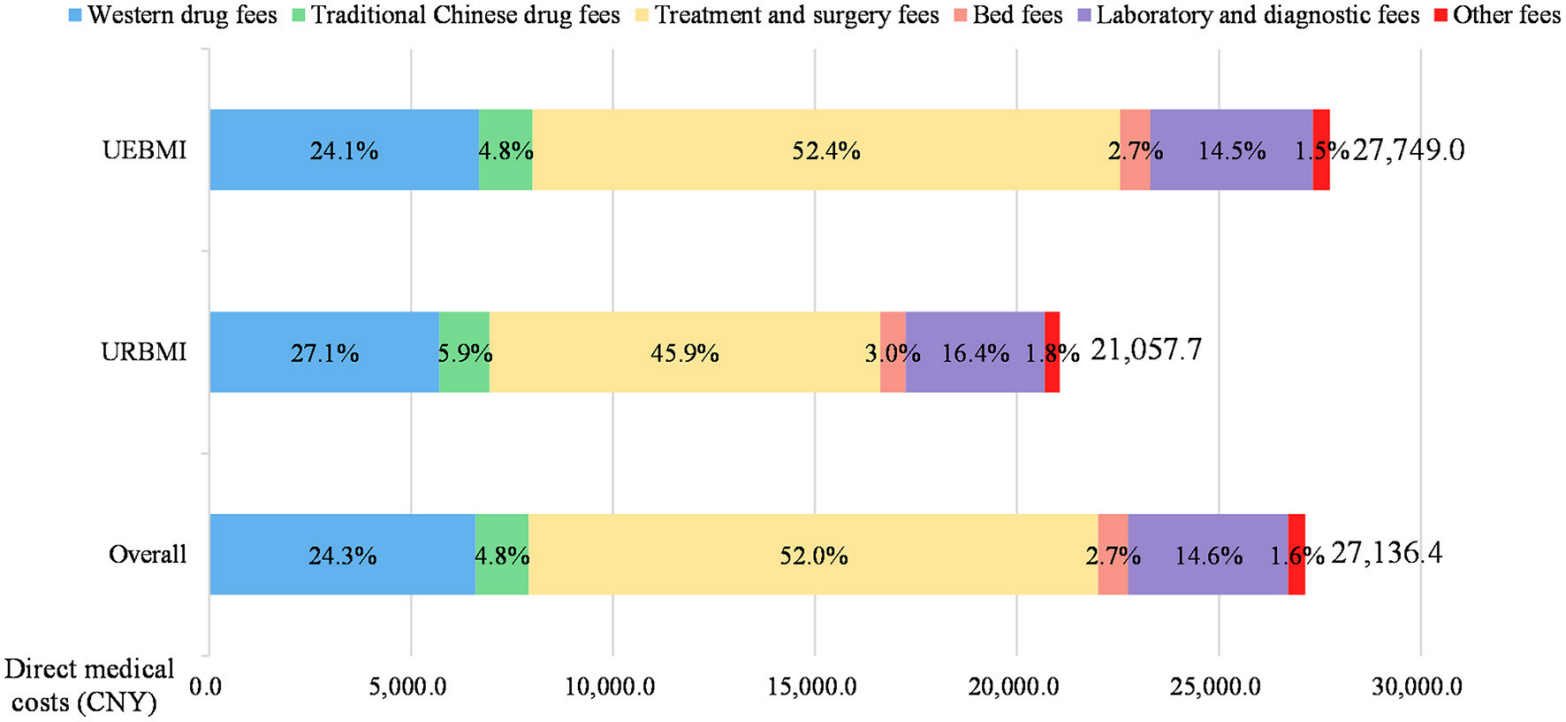
Composition of direct medical costs of ischemic heart disease patients. All costs were presented in 2012 Chinese Yuan (CNY). UEBMI, Urban Employee-based Basic Medical Insurance scheme; URBMI, Urban Resident-based Basic Medical Insurance scheme.

The mean direct medical costs per patient with IHD was CNY 27,136.4 (USD 4,298.8 in 2012), including CNY 26,283.2 (USD 4,163.7) for inpatient care and CNY 853.2 (USD 135.2) for outpatient care (see Table 2). The inpatient expenditures accounted for the majority of direct medical costs for IHD patients. The OOP expenses constituted 30.3% of the total direct medical costs. The percentage of OOP expenses out of inpatient costs (29.6%) was much lower than the OOP percentage out of outpatient expenditures (53.8%). Regarding the cost composition, the largest contributor of direct medical cost was treatment and surgery fees (52.0%), followed by western drug fees (24.3%), laboratory and diagnostic fees (14.6%), traditional Chinese drug fees (4.8%), bed fees (2.7%) and other fees (1.6%) (see Figure 1).

**Table 2:** Direct medical costs per patient with ischemic heart disease per year by type of insurance.

**Compositions**	**Overall**	**UEBMI**	**URBMI**	***P*-value**
No. patients	58,357	53,014	5,343	
**Total direct medical costs**				
Mean (CNY)	27,136.4	27,749.0	21,057.7	*P* < 0.001
SD	35,128.6	35,700.9	28,130.0	
**Out-of-pocket expenses**				
Percentage of direct medical costs (%)	30.3	28.7	52.0	
Mean (CNY)	8,235.1	7,961.6	10,948.9	*P* < 0.001
SD	12,412.7	11,907.1	16,371.5	
**Direct inpatient costs**				
No. patients having hospitalization	58,357 (100.0)	53,014 (100.0)	5,343 (100.0)	
Mean (CNY)	26,283.2	26,874.0	20,421.0	*P* < 0.001
SD	34,847.6	35,432.6	27,726.2	
**Out-of-pocket expenses**				
Percentage of inpatient costs (%)	29.6	27.9	51.5	
Mean (CNY)	7,775.3	7,499.7	10,510.3	*P* < 0.001
SD	12,163.2	11,667.6	16,030.1	
**Direct outpatient costs**				
No. patients visiting outpatient	28,378 (48.6)	26,552 (50.1)	1,826 (34.2)	
Mean (CNY)	853.2	875.0	636.6	*P* < 0.001
SD	1,279.4	1,271.3	1,337.5	
**Out-of-pocket expenses**				
Percentage of outpatient costs (%)	53.8	52.7	68.9	
Mean (CNY)	459.3	461.4	438.6	*P* < 0.001
SD	887.8	866.4	1,077.0	

*P*-values were based on Kruskal-Wallis Rank Sum Test; All costs were based on a constant 2012 Chinese Yuan (CNY); UEBMI, Urban Employee-based Basic Medical Insurance scheme; URBMI, Urban Resident-based Basic Medical Insurance scheme; CNY: Chinese Yuan; SD, standard deviation.

The comparison of costs and its composition between two different health insurance schemes was shown in Table 2 and Figure 1. The direct medical costs of IHD among patients in the UEBMI subgroup (CNY 27,749.0) (USD 4,395.9) was higher than patients in the URBMI subgroup (CNY 21,057.7) (USD 3,335.9). But the proportion of OOP expenditures out of direct medical costs for the UEBMI beneficiaries (28.7%) was approximately half of the OOP percentage for the URBMI beneficiaries (52.0%) (see Table 2). Furthermore, both inpatient costs and outpatient costs of IHD [CNY 26,874.0 (USD 4,257.3) and CNY 875.0 (USD 138.6)] among the UEBMI enrollees were significantly higher than those among [CNY 20,421.0 (USD 3,235.0) and CNY 636.6 (USD 100.8)] the URBMI enrollees (*P* < 0.001). In terms of cost composition, the treatment and surgery fees accounted for the largest proportion of direct medical costs for both the UEBMI enrollees (52.4%) and the URBMI enrollees (45.9%) (see Figure 1). The UEBMI group had higher proportion of treatment and surgery fees, but lower proportion of western drug fees, traditional Chinese drug fees, laboratory and diagnostic fees, bed fees and other fees than the URBMI group.

### Time trends of direct medical costs and out-of-pocket expenses by insurance type

**Figure 2:**
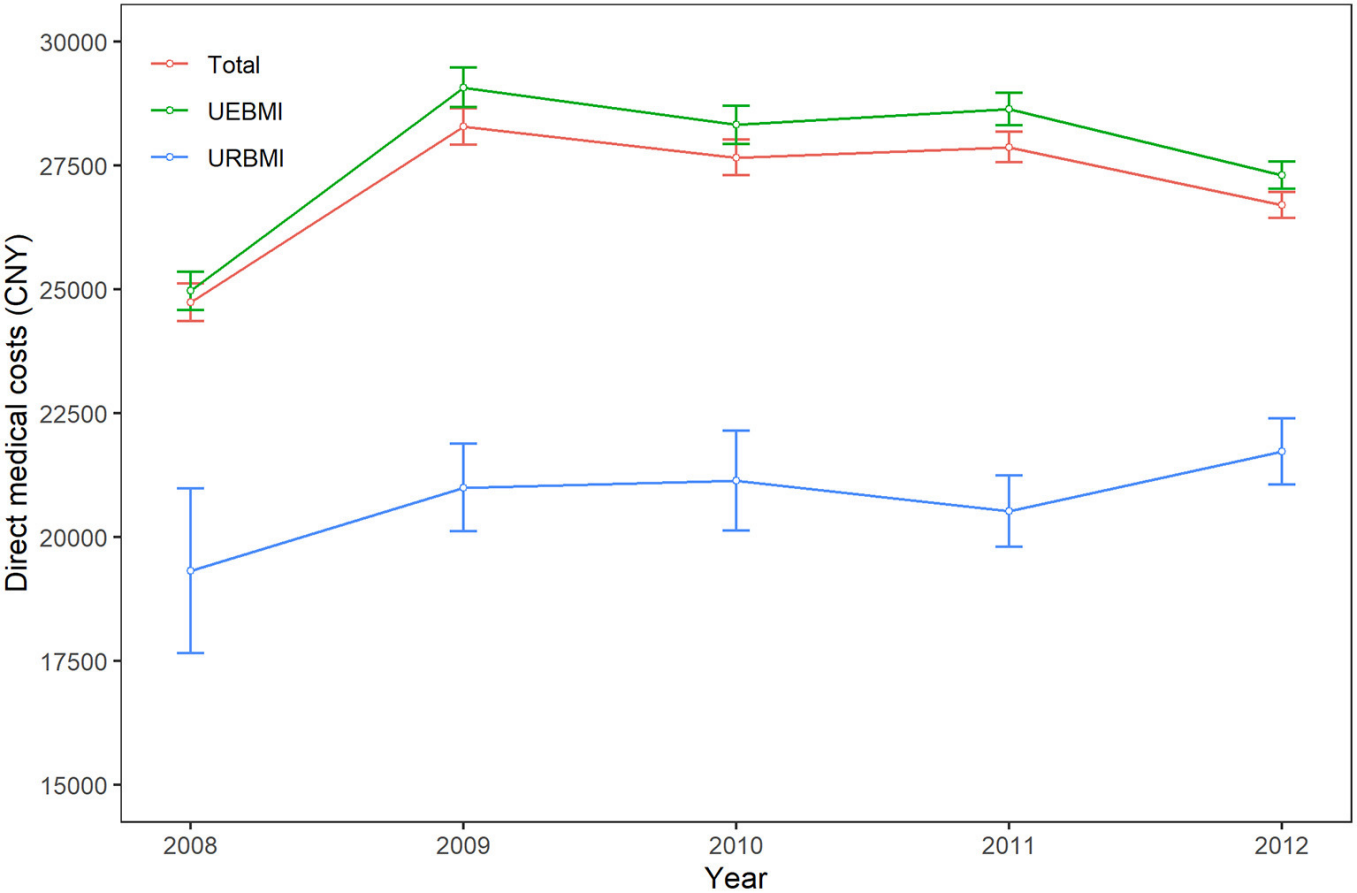
Time trends of direct medical costs of ischemic heart disease patients. All costs were reported on 2012 Chinese Yuan (CNY). UEBMI, Urban Employee-based Basic Medical Insurance; URBMI, Urban Resident-based Basic Medical Insurance.

As shown in Figure 2, the direct medical costs for all patients with IHD increased firstly from CNY 24,736.0 (USD 3,918.6) in 2008 to CNY 28,282.0 (USD 4,480.3) in 2009. However, there was a turning point in 2009 and then the direct medical costs decreased 1.9% per year during the period of 2009–2012.

**Figure 3:**
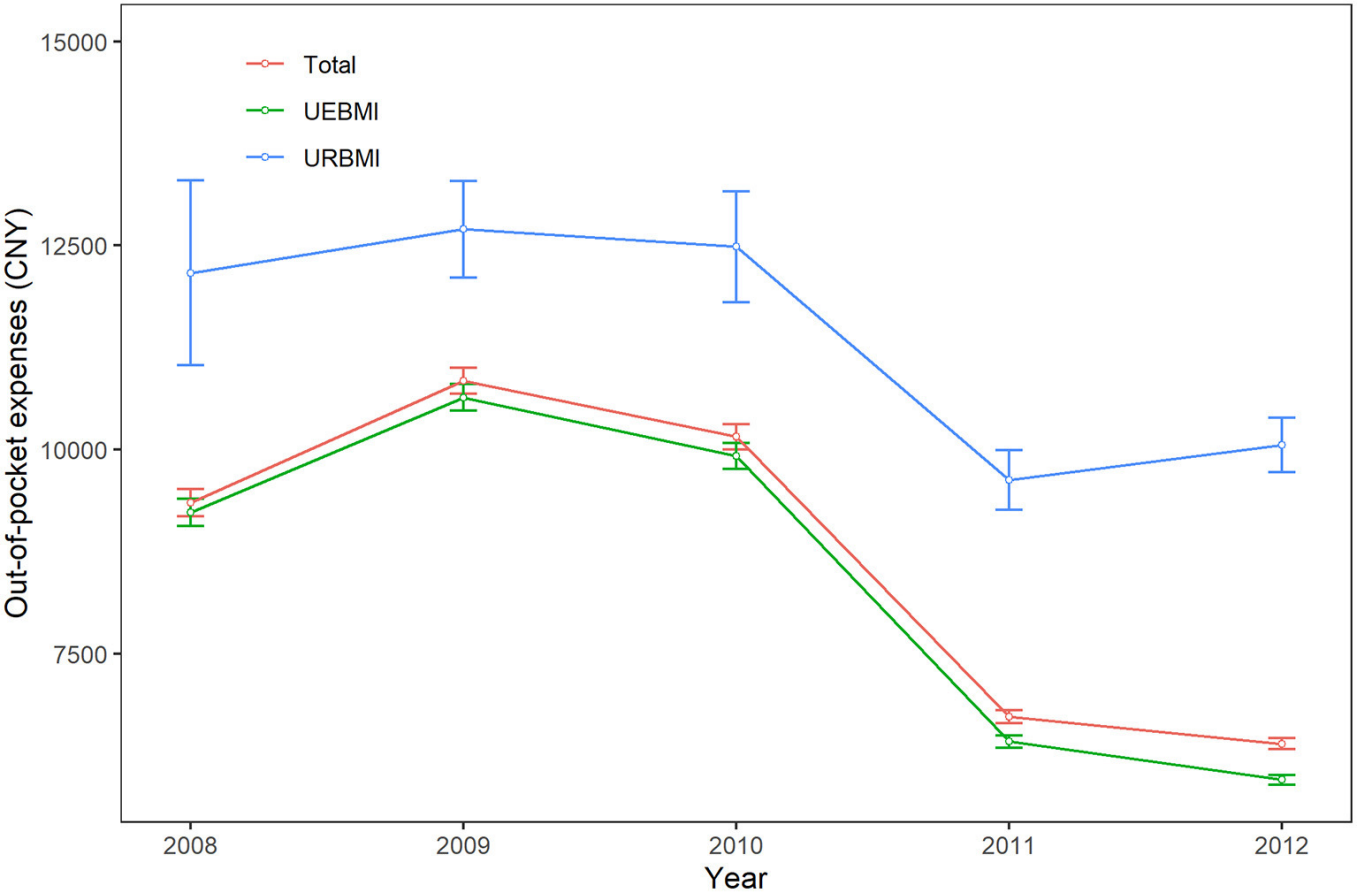
Time trends of out-of-pocket expenses of ischemic heart disease patients. All costs were reported on 2012 Chinese Yuan (CNY). UEBMI, Urban Employee-based Basic Medical Insurance; URBMI, Urban Resident-based Basic Medical Insurance.

The time trends of OOP expenses were similar to the direct medical costs. The OOP expenses of all IHD patients increased from CNY 9,351.1 (USD 1,481.4) in 2008 to CNY 10,840.9 (USD 1,717.4) in 2009 (see Figure 3) and there was also a turning point in 2009. The OOP expenses of all sample decreased during the period of 2009–2012.

When comparing two health insurance schemes, the direct medical costs for UEBMI patients initially increased from CNY 24,967.9 (USD 3,955.3) in 2008 to CNY 29,070.9 (USD 4,605.3) in 2009 and then decreased 2.1% per year during the period of 2009–2012. On the contrary, the direct medical costs of IHD for URBMI patients increased 3.0% per year from CNY 19,325.8 (USD 3,061.5) in 2008 to CNY 21,730.9 (USD 3,442.5) in 2012. The time trends of OOP expenses among UEBMI subgroup and URBMI subgroup were similar.

### Patient characteristics associated with direct medical costs and out-of-pocket expenses by type of insurance

Direct medical costs and OOP expenses for patients with IHD across different types of insurance significantly differed by gender, age, hospital level, LOS and comorbidities (all *P* < 0.001) (see Table 3). For the overall samples, UEBMI subgroup and URBMI subgroup, male patients incurred higher direct medical costs and OOP expenses than female patients. Patients aged 60–69 years old had the highest direct medical costs for overall samples and two insurance subgroups. And patients aged 18–59 years old had the highest OOP expenses for overall samples and the UEBMI subgroup, but those aged 60–69 years old had the highest OOP expenses for the URBMI subgroup.

**Table 3:** Ischemic heart disease patient characteristics associated with direct medical costs and out-of-pocket expenses.

**Patient characteristics** **No. patients**	**Direct medical costs**							**Out-of-pocket expenses**						
	**Overall**		**UEBMI**		**URBMI**		***P*** **-value**	**Overall**		**UEBMI**		**URBMI**		***P*** **-value**
	***n*** = **58,357**		***n*** = **53,014**		***n*** = **5,343**			***n*** = **58,357**		***n*** = **53,014**		***n*** = **5,343**		
	**Mean**	**SD**	**Mean**	**SD**	**Mean**	**SD**		**Mean**	**SD**	**Mean**	**SD**	**Mean**	**SD**	
**Gender**							*P* < 0.001							*P* < 0.001
Female	22,234.1	28,941.0	22,739.7	29,382.2	18,357.6	24,968.8		6,402.3	9.988.05	6,017.8	9,201.7	9,348.6	14,342.9	
Male	32,350.5	40,037.1	32,796.7	40,474.9	26,060.0	32,622.3		10,183.9	14,299.6	9,919.3	13,845.1	13,913.6	19,237.7	
**Age group**							*P* < 0.001							*P* < 0.001
18≤Age<59	29,574.7	36,748.8	29,949.3	37,048.8	23,907.0	31,354.0		10,981.2	14,937.5	10,864.1	14,691.5	12,752.2	18,182.6	
60≤Age<69	30,148.6	39,034.3	30,634.5	39,710.2	25,346.3	31,195.4		9,729.9	13,684.7	9,305.2	13,041.7	13,927.7	18,384.8	
70≤Age<79	28,286.4	35,902.4	28,880.3	36,445.3	22,274.0	29,189.7		8,348.0	12,301.2	8,007.5	11,669.4	11,795.7	17,080.7	
≥80	21,912.8	28,898.3	22,633.3	29,510.2	16,115.1	22,584.3		5,373.2	8,876.0	5,100.2	8,250.7	7,570.3	12,634.1	
**Hospital level**							*P* < 0.001							*P* < 0.001
Primary	7,804.5	10,128.6	7,904.0	9,986.9	7,206.1	10,929.1		1,409.2	2,391.8	1,280.2	1,829.2	2,184.5	4,395.3	
Secondary	12,258.0	17,608.3	12,516.3	18,135.6	10,182.6	12,405.7		2,724.9	4,109.2	2,563.0	3,956.6	4,026.1	4,988.3	
Tertiary	35,034.0	39,015.7	35,453.2	39,448.8	30,053.7	33,044.7		11,111.2	14,041.6	10,644.1	13,389.1	16,661.4	19,405.9	
**Length of stay (days)**							*P* < 0.001							*P* < 0.001
Days < 15	20,118.4	25,935.9	20,611.8	26,357.3	15,478.5	21,017.5		6,934.9	10,559.3	6,773.3	10,284.8	8,454.5	12,758.8	
15≤Days<30	34,271.3	36,390.3	34,803.2	36,808.4	28,642.6	31,089.8		10,013.1	13,855.7	9,579.7	13,220.4	14,600.0	18,742.2	
Days ≥ 30	56,784.1	57,898.8	57,437.1	58,688.0	48,564.6	46,109.8		12,722.5	17,545.4	11,939.5	16,334.0	22,577.5	26,853.6	
**Comorbidity**							*P* < 0.001							*P* < 0.001
None	28,313.4	36,226.2	28,984.1	36,844.0	21,808.4	28,777.0		8,940.1	13,018.5	8,674.4	12,507.4	11,516.8	16,992.9	
Hypertension	22,411.2	30,020.8	22,833.8	30,432.4	18,111.9	25,066.3		5,861.7	9,718.8	5,570.2	9,196.5	8,827.3	13,619.0	
Diabetes mellitus	30,953.5	38,478.9	31,637.9	39,055.6	22,325.0	28,973.4		8,232.6	12,425.0	7,997.0	12,027.2	11,203.0	16,380.0	

*P*-values are based on the Friedman's two-way non-parametric analysis of variance test; All costs were based on a constant 2012 Chinese Yuan (CNY); UEBMI, Urban Employee-based Basic Medical Insurance scheme; URBMI, Urban Resident-based Basic Medical Insurance scheme; SD, standard deviation; ICU, Intensive Care Unit; PCI, Percutaneous Coronary Intervention.

In addition, among the overall samples and all subgroups, the mean direct medical costs and OOP expenses of IHD patients recruited in tertiary hospitals were far more than those of patients staying in primary hospitals. Patients with LOS longer than 30 days had higher direct medical costs and OOP expenses than patients with shorter LOS. Patients having diabetes mellitus had higher direct medical cost than those with hypertension.

### Associated factors of direct medical costs and out-of-pocket expenses

This study found that the types of insurance, PCI operation, gender, age, hospital levels, ICU admission, LOS, and comorbidities (hypertension and diabetes mellitus) were significantly associated with direct medical costs and OOP expenses of IHD (see Table 4). Compared with patients covered by the URBMI scheme, the direct medical costs for IHD patients insured by the UEBMI scheme were significantly higher (CNY 692.5) (USD 109.7), and their OOP expenses were significantly lower (CNY 5,416.1) (USD 858.0) (*P* < 0.001), after controlling for other covariates (*P* < 0.001).

**Table 4:** Factors associated with direct medical costs and out-of-pocket expenses of ischemic heart disease (EEE model).

**Associated factors**	**Direct medical costs**			**Out-of-pocket expenses**		
	***n*** = **58,357**			***n*** = **58,357**		
	**Coef**.	**Adjusted std. err**.	**Marginal effect**	**Coef**.	**Adjusted std. err**.	**Marginal effect**
**Gender**						
Female (Reference)						
Male	0.118^***^	0.006	2,962.8	0.191^***^	0.008	1,464.7
**Age group**						
18≤Age<59 (Reference)						
60≤Age<69	0.001	0.011	3.4	−0.122^***^	0.013	−918.6
70≤Age<79	−0.013	0.010	−327.0	−0.192^***^	0.012	−1,460.3
≥80	−0.047^***^	0.010	−1,159.3	−0.326^***^	0.013	−2,349.4
**Insurance type**						
URBMI (Reference)						
UEBMI	0.028^***^	0.009	692.5	−0.577^***^	0.014	−5,416.1
**Hospital level**						
Primary (Reference)						
Secondary	0.265^***^	0.008	7,093.3	0.148^***^	0.015	1,192.9
Tertiary	0.870^***^	0.011	19,006.6	0.842^***^	0.016	5,505.5
ICU admission	0.584^***^	0.008	76,882.9	1.500^***^	0.105	21,401.4
PCI operation	1.432^***^	0.013	51,211.0	1.665^***^	0.012	17,893.0
**Length of stay(days)**						
Days < 15 (Reference)						
15≤Days<30	1.707^***^	0.087	16,432.5	0.436^***^	0.010	3,705.0
Days ≥ 30	1.509^***^	0.009	53,216.7	1.005^***^	0.015	10,763.1
**Year**						
Year 2008 (Reference)						
Year 2009	0.112^***^	0.011	2,888.0	0.087^***^	0.015	688.4
Year 2010	0.128^***^	0.011	3,321.7	0.043^***^	0.016	339.1
Year 2011	0.151^***^	0.010	3,898.9	−0.230^***^	0.014	−1,694.9
Year 2012	0.152^***^	0.010	3,908.1	−0.240^***^	0.013	−1,777.0
**Comorbidity**						
None (Reference)						
Hypertension	−0.073^***^	0.006	−1,806.4	−0.127^***^	0.009	−951.4
Diabetes mellitus	0.045^***^	0.010	1,139.8	−0.043^***^	0.014	−329.4
λ	0.265^***^	0.009		0.160^***^	0.010	
θ1	0.575^***^	0.011		0.819^***^	0.019	
θ2	1.829^***^	0.018		1.654^***^	0.031	

The Extended Estimating Equations (EEE) model estimates were reported in the table; Coef., coefficient; Adjusted std. err. are standard errors adjusted for clustering at the patient level; UEBMI, Urban Employee-based Basic Medical Insurance scheme; URBMI, Urban Resident-based Basic Medical Insurance scheme; ICU, Intensive Care Unit; PCI, Percutaneous Coronary Intervention;

*** *P* < 0.001.

Male patients had significantly higher direct medical costs and OOP expenses than their female counterparts (*P* < 0.001). Compared with patients aged 18–59, patients 60–69 and 70–79 had CNY 918.6 (USD 145.5) and CNY 1,460.3 (USD 231.3) lower OOP expenses (*P* < 0.001), and patients aged 80 and above had CNY 1,159.3 (USD 183.7) lower direct medical costs and CNY 2,349.4 (USD 372.2) lower OOP expenses (*P* < 0.001). Patients having PCI operation and ICU admission incurred significantly higher direct medical costs and OOP expenses (*P* < 0.001). Compared to patients treated in primary hospitals, IHD patients staying in secondary hospitals had CNY 7,093.3 (USD 1,123.7) higher direct medical costs and CNY 1,192.9 (USD 189.0) higher OOP expenses (*P* < 0.001), and patients staying in tertiary hospitals had CNY 19,006.6 (USD 3,010.9) higher direct medical costs and CNY 5,505.5 (USD 872.2) higher OOP expenses (*P* < 0.001). In comparison with LOS of <15 days, patients with the LOS between 15 and 30 days had CNY16,432.5 (USD 2,603.2) higher direct medical costs and CNY 3,705.0 (USD 586.9) higher OOP expenses (*P* < 0.001), and patients with the LOS of more than 30 days had CNY53,216.7 (USD 8,430.4) higher direct medical costs and CNY 10,763.1 (USD 1,705.0) higher OOP expenses(*P* < 0.001). Also, IHD patients with hypertension incurred lower direct medical costs and lower OOP expenses (*P* < 0.001), but patients with diabetes mellitus had higher direct medical costs and lower OOP expenses (*P* < 0.001).

The results of the heterogeneity analysis indicated that hospital level had an effect on the association between types of insurance and direct medical costs, while hospital level and LOS had an effect on the association between types of insurance and OOP expenses. Details on the results of heterogeneity analysis in this study are shown in Appendix A.

## Discussion

This was a retrospective study using a large sample of 58,357 urban patients with IHD in Guangzhou City, Southern China. This research found that the direct medical costs per patient with IHD were CNY 27,136.4 (USD 4,298.8), which included CNY 26,283.2 (USD 4,163.7) for inpatients care and CNY 853.2 (USD 135.2) for outpatient care. The treatment and surgery fees accounted for the largest part of direct medical costs (52.0%) for IHD patients. The direct medical costs of IHD patients covered by the UEBMI schemes (CNY 27,749.0) (USD 4,395.9) were higher than those of patients covered by the URBMI schemes (CNY 21,057.7) (USD 3,335.9). The direct medical costs and OOP expenses for all patients increased from 2008 to 2009, and then decreased during the period of 2009–2012. The time trends of direct medical costs between the UEBMI and URBMI patients were different during the period of 2008–2012. This study also discovered that the type of medical insurance, gender, age, hospital levels, ICU admission, PCI operation, LOS, and comorbidities (hypertension and diabetes mellitus) were significantly associated with total direct medical costs and OOP expenses of IHD. The UEBMI enrollees had higher direct medical costs but had lower OOP expenses than the URBMI enrollees. This was the first study to estimate the direct medical costs and OOP expenses of IHD for the urban population from all levels of hospitals using an entire city's claims datasets, including both inpatient and outpatient costs. Different from previous China-based studies, this research also compared the medical expenses and OOP expenses of IHD patients between two different urban health insurance schemes and examined the time trend of direct medical costs and OOP expenses in China.

### Comparison of costs with previous studies in other countries

Compared with studies in other countries, we found that there were plenty of disparities in costs estimation results for IHD patients. In this study, the direct medical costs of IHD were CNY 27,136.4 (USD 4,298.8 in 2012), much lower than the results in the US (USD 22,921.0 in 2012) (21) and England (USD 4,501.4 in 2012) (22), but higher than the expenses in Poland (USD 3,046.3 in 2012) (23), Korea (USD 2,099.04 in 2012) (27), Brazil (USD 1,452.4 in 2012) (24), Cameroon (USD 2,227.4 in 2012) (25), and Iran (USD 1,507.0 in 2012) (26). It was widely recognized that the developing countries had poorer health service capacity and financing situation (46), which might be related to the variation of health expenditures in different countries. Among all literature compared in this study, there were also great disparities in study design, sources of data, and type of costs, which may also lead to the different costs between this study and those of previous studies in other countries.

### Comparison of costs with previous studies in China

Comparing with prior studies conducted in China, the average direct medical costs of IHD in this study (USD 4,298.9 in 2012) were higher than that of Le et al.'s study (USD 1,203.0 in 2012) (30) and Wang et al.'s study (USD 976.6 in 2012) (28). Patients in these studies (28, 30) were recruited from rural areas, in which most of the hospitals were township hospitals or primary care units without advanced medical equipment. Patients in rural areas were less likely to receive prompt PCI and reperfusion therapy compared to urban patients (47), which may lower the direct medical costs. In addition, Wang et al.'s study (28) only evaluated the hospitalization costs of IHD, but our study included both inpatient and outpatient care that might induce higher direct medical costs of IHD.

However, the direct medical costs in this study were lower than that of Ding et al.'s study (USD 6,481.0 in 2012) (29). A possible explanation was that Ding et al. study (29) recruited samples from only one tertiary hospital, but our study included patients with IHD from all different levels of hospitals. Patients in tertiary hospitals usually incurred higher expenses than those in secondary and primary hospitals (48), which may induce higher costs.

### Comparison of costs composition

In terms of costs composition, treatment and surgery fees occupied the largest proportion of direct medical costs for IHD patients (52.0%) in this study. This may be due to substantially higher cost of invasive therapeutic procedures (49). This result was consistent with the findings of prior studies (26, 50), indicating that surgical procedures were the main sources of hospitalization costs. The treatment of IHD included surgical or other advanced medical procedures, and post-operative care (40). Darba et al. (26) reported that surgery costs accounted for more than 50% of direct medical costs. Khan et al. (50) noted that surgical procedures expenses occupied 75.99% of total expenditures on treatment of IHD. The higher treatment costs were related with surgical interventions, including PCI (40). The regression result of this study also found that patients who received PCI operation incurred significantly higher direct medical costs. This was in agreement with a previous study (38), indicating patients with PCI treatment were associated with higher expenditures.

The spending on medical consumables such as heart stent implantation was also a critical component for treatment and surgery fees. Evidence in previous China-based studies (29, 51) illustrated that the expenditures on medical consumable had occupied the large proportion of total expenses, accounting for more than 30% of total medical costs. The increasing usage of medical consumables became the main factors associated with direct medical expenses growth in China (52).

The medication costs took up the second largest part of direct medical costs for IHD, including Western drug fees (24.3%) and traditional Chinese drug fees (4.8%). The proportion of total medication costs (29.1%) was similar to the figure in Poland (30%) (23), but lower than the results in Brazil (51%) (24). The higher percentage of drug costs in Brazil was due to the entry of new medicines with higher prices into the market and the reduction on costs of medical consumables such as orthotics and prostheses (stents) (24). Pharmacological treatment of IHD including beta-blocker, angiotensin-converting enzyme inhibitors/angiotensin receptor blockers, antiplatelet drugs, and statins (53), and this was important for long-term treatment on IHD patients (54). The patterns of medication use, including multidrug therapy of 1 to 2 medications and multidrug therapy of 3–4 medications, were different across countries (55). As a result, the variation in patterns of medication use in different countries might be also related to the difference in medication costs. Different from previous studies in other countries, this research pointed out that Traditional Chinese medicine also played an important role in the management of IHD. It had been proved that the Traditional Chinese medicines combined with antiplatelet drugs and other conventional Western medicines was effective in the perioperative treatment of patients with IHD undergoing PCI therapy (56). Furthermore, the Traditional Chinese medicine can be applicable to the management of disease recovery and rehabilitation phases (57).

### Difference in costs between two insurance schemes

Different from previous China-based studies, discrepancies in direct medical costs and OOP expenses of patients with IHD across two types of basic health insurance schemes were observed in this study for the first time. The direct medical costs of IHD patients enrolled in UEBMI schemes (CNY 27,749.0) (USD 4395.9) were higher than those of patients covered by the URBMI schemes (CNY 21,057.7) (USD 3335.9). But the proportion of OOP expenses out of direct medical costs for the UEBMI patients (28.7%) was approximately half of the OOP percentage for the URBMI patients (52.0%). The regression findings also suggested that UEBMI enrollees had significantly higher direct medical costs with IHD including both inpatient and outpatient care, but had lower OOP expenses than URBMI enrollees.

These findings were in line with previous studies estimating discrepancies in expenditures of other diseases under different types of social health insurance (58–60). UEBMI enrollees with IHD incurred significantly higher direct medical costs than the URBMI enrollees. There were two explanations for this phenomenon. At first, the UEBMI scheme provided more generous benefit packages, higher reimbursement rates and wider service coverage (16), which could induce UEBMI patients to seek for more comprehensive health services and thus incurred higher direct medical costs and lower OOP expenses. Second, the UEBMI enrollees often had a stable source of income and better accessibility to health services (59), while the URBMI enrollees were usually the unemployed, students and children with poorer financial situations (61). As a result, the URBMI patients with lower income could be more conservative when using medical services and drugs (62), which might lead to lower spending. Since the variation in direct medical costs and OOP expenses of patients with IHD across these two basic medical insurance schemes, it was important to consolidate health insurance schemes and achieve universal health coverage for Chinese urban population.

This study also found that the time trends of direct medical costs between the UEBMI and URBMI patients were different during the period of 2008–2012 for the first time. The direct medical costs of URBMI patients kept increasing during the period of 2008–2012. However, the direct medical costs of UEBMI enrollees had decreased since 2009. This was probably associated with a new policy on improving outpatient benefit packages only for UEBMI enrollees initiated in 2009 (63), which may lead to higher outpatient visits. Previous studies had proved that the increasing outpatient visits and outpatient reimbursement rates were significantly associated with lower inpatient costs (64, 65), and then the total direct medical costs including both outpatient and inpatient care would decrease.

### Associated factors of direct medical costs and out-of-pocket expenses

#### Age

In this study, we demonstrated that those aged 80 and above had significantly lower direct medical costs and lower OOP expenses than those aged under 60. Previous studies found that older patients with IHD incurred higher medical costs than younger patients (24, 29). However, our finding was similar to another China-based study (28), which also found that younger patients aged 60 years and below had significantly higher hospitalization costs than those aged 80 years above. This may be due to the variation in sample selection among different studies.

#### Gender

In consistent with previous studies (22, 24, 28, 29), we found that male patients had higher direct medical costs than female patients. The possible reasons might be the higher IHD burden (13), higher morbidity of IHD (8), and more consumption of drinking and smoking (66) for male patients than female patients, and thus male patients may suffer from more serious cardiovascular disease and incurred higher expenditures.

#### Hospital level

Patients treated in higher levels of hospitals incurred significantly higher direct medical costs in our study, consistent with previous research in China (36, 37, 48). Due to having more medical resources in tertiary hospitals, most of the patients with acute or complicated diseases tended to be treated in tertiary hospitals rather than primary hospitals (67). As a result, tertiary hospitals that were mainly responsible for the diagnosis and treatment of acute, critical, and complicated diseases (68), would incur higher medical expenses.

#### LOS

In accordance with other literature (28, 29), longer LOS was significantly associated with higher direct medical costs in this study. The average LOS in this study was 16.1 days, longer than the figures reported in Iran (10.34 days) (26). It was also longer than the figures mentioned in previous China-based studies in Liaoning province (8.89 days) (28) and Xi'an city (4.0 days) (29). A possible reason for this phenomenon was that the LOS reported in Iran (26) and Xi'an city (29) were estimated using LOS per admission instead of LOS per patient. Since ~21% of patients with an IHD diagnosis would be rehospitalized in 1 year (21), the LOS per patient per year in our study including several admissions might be much longer. Another China-based study in Liaoning province also reported the LOS per patient (28), but it only recruited patients from township hospitals where their patients might not be critically ill and then induce lower LOS than our study. Our regression analysis showed that the direct medical costs increased with longer LOS. Similar as this result, Brouwer et al. (40) also illustrated that the higher inpatient costs were associated with longer hospitalization periods. Patients with longer LOS might suffer from more severe diseases and probably more serious comorbidities (69), which could induce higher costs. In order to reduce the LOS, less ill patients and those recovering patients who can be treated by both outpatient and inpatient services could be transferred to outpatient treatment (70).

There were some limitations in this research. First, we only included urban patients with IHD insured by two urban basic medical insurance schemes, and excluded rural residents, which might cause selection bias. Second, the study only estimated the direct medical costs of IHD and did not evaluate the indirect costs such as informal care and loss of productivity. Third, the dataset was a little bit old due to administrative restrictions on data availability, which may not reveal the IHD costs and differences at present. Future studies could consider using a more recent claims dataset, as well as including people covered by all types of insurance, and examining the indirect costs for a more comprehensive evaluation of IHD costs. Fourth, individual factors and disease factors, which were more critical to determine the economic burden, were not introduced in this study because such data were not available in the claims dataset. Finally, the specificity of IHD, such as case characteristics and treatment methods, was not reflected in our analysis due to the unavailability of such data in the claims dataset. Further studies could consider adding case characteristics and treatment methods to examine the determinants of IHD costs.

## Conclusion

The direct medical costs and OOP expenses of patients with IHD for Chinese urban population were high and different across two basic health insurance schemes. Types of health insurance, gender, age, hospital levels, LOS, ICU admission, PCI operation and comorbidities were significantly associated with the direct medical costs and OOP expenses of IHD. Therefore, policymakers should aim to reduce disparities across different health insurance schemes in terms of benefit packages and narrow the economic burden gap among IHD patients covered by different insurance schemes.

## Data availability statement

The datasets used and analyzed during the current study are available from the corresponding author on reasonable request.

## Ethics statement

The studies involving human participants were reviewed and approved by the Institutional Review Board of the School of Public Health, Sun Yat-sen University, China (Approval No. 2022025). Written informed consent for participation was not required for this study in accordance with the national legislation and the institutional requirements.

## Author contributions

Conceptualization, methodology, resources, supervision, project administration, and funding acquisition: HZ. Software and formal analysis: PX. Validation: HZ and DZ. Investigation: PX, XL, and FG. Data curation and writing—original draft preparation: HZ and PX. Writing—review and editing: HZ, DZ, and XL. All authors have read and agreed to the submitted version of the manuscript.
