# Supplementary material for: Direct medical costs of ischemic heart disease in urban Southern China: a 5-year retrospective analysis of an all-payer health claims database in Guangzhou City
Source: Front Public Health. 2023 May 9;11:1146914. doi: 10.3389/fpubh.2023.1146914 (PMC10203198; doi:10.3389/fpubh.2023.1146914)
Supplement: Supplementary file 2 [file Table_1.docx]

Appendix A

**Table A1** The association between types of insurance and direct medical costs among different hospital level subgroups.

| **Associated Factors** | **Primary hospital level** | | | **Secondary hospital level** | | | **Tertiary hospital level** | | |
| --- | --- | --- | --- | --- | --- | --- | --- | --- | --- |
|  | Coef. | Adjusted Std.err. | Marginal Effect | Coef. | Adjusted Std.err. | Marginal Effect | Coef. | Adjusted Std.err. | Marginal Effect |
| **Gender** |  |  |  |  |  |  |  |  |  |
| Female (Reference) |  |  |  |  |  |  |  |  |  |
| Male | 0.056*** | 0.015 | 1,141.5 | 0.125*** | 0.013 | 2,557.1 | 0.135*** | 0.008 | 3,625.8 |
| **Age group** |  |  |  |  |  |  |  |  |  |
| 18≤Age<59 (Reference) | 0.056** | 0.025 | 1,146.5 | 0.017 | 0.026 | 354.1 | -0.001 | 0.012 | -26.7 |
| 60≤Age<69 | 0.027 | 0.022 | 548.7 | 0.020 | 0.024 | 417.5 | -0.013 | 0.011 | -343.9 |
| 70≤Age<79 | -0.010 | 0.020 | -210.8 | -0.029 | 0.023 | -591.1 | -0.049*** | 0.012 | -1,303.7 |
| ≥80 | 0.056** | 0.025 | 1,146.5 | 0.017 | 0.026 | 354.1 | -0.001 | 0.012 | -26.7 |
| **Insurance type** |  |  |  |  |  |  |  |  |  |
| URBMI(Reference) |  |  |  |  |  |  |  |  |  |
| UEBMI | 0.107*** | 0.019 | 2,113.0 | 0.084*** | 0.016 | 1,678.7 | 0.007 | 0.013 | 196.1 |
| **ICU admission** | 2.857*** | 0.675 | 109,800.5 | 2.331*** | 0.359 | 110,563.2 | 2.339*** | 0.156 | 90,036.5 |
| **PCI operation** | 2.075*** | 0.045 | 61,000.0 | 1.944*** | 0.031 | 60,558.0 | 1.684*** | 0.015 | 55,308.6 |
| **Length of stay(days)** |  |  |  |  |  |  |  |  |  |
| Days<15 (Reference) |  |  |  |  |  |  |  |  |  |
| 15≤Days<30 | 0.331*** | 0.033 | 7,146.0 | 0.536*** | 0.027 | 12,303.3 | 0.614*** | 0.011 | 17,579.4 |
| Days≥30 | 1.037*** | 0.061 | 26,739.1 | 1.349*** | 0.038 | 40,984.8 | 1.636*** | 0.030 | 55,019.8 |
| **Year** |  |  |  |  |  |  |  |  |  |
| Year 2008 (Reference) |  |  |  |  |  |  |  |  |  |
| Year 2009 | 0.107*** | 0.026 | 2,236.4 | 0.115*** | 0.020 | 2,439.3 | 0.098*** | 0.014 | 2,662.9 |
| Year 2010 | 0.125*** | 0.027 | 2,615.1 | 0.144*** | 0.020 | 3,074.6 | 0.098*** | 0.014 | 2,643.2 |
| Year 2011 | 0.154*** | 0.028 | 3,217.1 | 0.245*** | 0.021 | 5,290.8 | 0.098*** | 0.013 | 2,644.1 |
| Year 2012 | 0.219*** | 0.031 | 4,584.1 | 0.255*** | 0.021 | 5,469.9 | 0.091*** | 0.012 | 2,459.6 |
| **Comorbidity** |  |  |  |  |  |  |  |  |  |
| None (Reference) |  |  |  |  |  |  |  |  |  |
| Hypertension | -0.031** | 0.014 | -628.3 | -0.092*** | 0.012 | -1,849.8 | -0.079*** | 0.009 | -2,082.6 |
| Diabetes mellitus | 0.011 | 0.024 | 231.2 | 0.046** | 0.019 | 964.0 | 0.046*** | 0.014 | 1,231.0 |
| Constant | -1.311*** | 0.083 |  | -1.465*** | 0.053 |  | -0.700*** | 0.020 |  |
| λ | 0.528*** | 0.072 |  | 0.260*** | 0.039 |  | 0.594*** | 0.021 |  |
| θ1 | 0.593*** | 0.047 |  | 0.647*** | 0.032 |  | 0.589*** | 0.013 |  |
| θ2 | 1.556*** | 0.075 |  | 1.884*** | 0.046 |  | 1.460*** | 0.025 |  |
| Observations | 7,100 |  |  | 13,381 |  |  | 37,876 |  |  |

Notes: The Extended Estimating Equations (EEE) model estimates were reported in the table; Coef., coefficient; Adjusted Std.err. are standard errors adjusted for clustering at the patient level; UEBMI, Urban Employee-based Basic Medical Insurance scheme; URBMI, Urban Resident-based Basic Medical Insurance scheme; ICU, Intensive Care Unit; PCI, Percutaneous Coronary Intervention; ***, *P*<0.001, **, *P* <0.01.

**Table A2** The association between types of insurance and out-of-pocket expenses among different hospital level subgroups.

| **Associated Factors** | **Primary hospital level** | | **Secondary hospital level** | | | | | **Tertiary hospital level** | | |
| --- | --- | --- | --- | --- | --- | --- | --- | --- | --- | --- |
|  | Coef. | Adjusted Std.err. | | Marginal Effect | Coef. | Adjusted Std.err. | Marginal Effect | Coef. | Adjusted Std.err. | Marginal Effect |
| **Gender** |  |  | |  |  |  |  |  |  |  |
| Female (Reference) |  |  | |  |  |  |  |  |  |  |
| Male | 0.177*** | 0.031 | | 934.1 | 0.174*** | 0.018 | 1,011.8 | 0.197*** | 0.009 | 1,742.4 |
| **Age group** |  |  | |  |  |  |  |  |  |  |
| 18≤Age<59 (Reference) |  |  | |  |  |  |  |  |  |  |
| 60≤Age<69 | 0.038 | 0.050 | | 206.9 | -0.083** | 0.037 | -474.6 | -0.150*** | 0.015 | -1,288.7 |
| 70≤Age<79 | -0.034 | 0.044 | | -180.6 | -0.135*** | 0.034 | -780.7 | -0.221*** | 0.014 | -1,930.7 |
| ≥80 | -0.156*** | 0.044 | | -803.4 | -0.273*** | 0.036 | -1,497.1 | -0.345*** | 0.016 | -2,867.1 |
| **Insurance type** |  |  | |  |  |  |  |  |  |  |
| URBMI (Reference) |  |  | |  |  |  |  |  |  |  |
| UEBMI | -0.288*** | 0.049 | | -1,704.3 | -0.462*** | 0.034 | -3,204.6 | -0.696*** | 0.017 | -7,779.6 |
| **ICU admission** | 2.405*** | 0.322 | | 35,988.9 | 2.115*** | 0.273 | 32,627.3 | 1.375*** | 0.106 | 21,300.9 |
| **PCI operation** | 2.363*** | 0.052 | | 20,383.1 | 2.159*** | 0.036 | 19,772.9 | 1.585*** | 0.010 | 19,993.7 |
| **Length of stay(days)** |  |  | |  |  |  |  |  |  |  |
| Days<15 (Reference) |  |  | |  |  |  |  |  |  |  |
| 15≤Days<30 | 0.331*** | 0.040 | | 1,906.1 | 0.433*** | 0.034 | 2,811.6 | 0.453*** | 0.012 | 4,445.2 |
| Days≥30 | 0.892*** | 0.083 | | 6,426.9 | 1.007*** | 0.054 | 8,419.0 | 1.043*** | 0.022 | 12,905.8 |
| **Year** |  |  | |  |  |  |  |  |  |  |
| Year 2008 (Reference) |  |  | |  |  |  |  |  |  |  |
| Year 2009 | 0.144*** | 0.056 | | 801.8 | 0.098*** | 0.029 | 589.4 | 0.073*** | 0.019 | 658.8 |
| Year 2010 | 0.076 | 0.053 | | 414.4 | 0.044 | 0.028 | 262.6 | 0.032 | 0.019 | 282.4 |
| Year 2011 | -0.048 | 0.050 | | -254.2 | -0.013 | 0.023 | -77.3 | -0.344*** | 0.017 | -2,846.7 |
| Year 2012 | 0.022 | 0.048 | | 120.2 | -0.045** | 0.021 | -261.0 | -0.363*** | 0.016 | -3,032.3 |
| **Comorbidity** |  |  | |  |  |  |  |  |  |  |
| None (Reference) |  |  | |  |  |  |  |  |  |  |
| Hypertension | -0.068** | 0.028 | | -357.7 | -0.159*** | 0.018 | -890.5 | -0.131*** | 0.012 | -1,132.2 |
| Diabetes mellitus | -0.088* | 0.048 | | -452.9 | -0.055** | 0.026 | -315.5 | -0.032* | 0.018 | -281.6 |
| Constant | -1.276*** | 0.088 | |  | -0.855*** | 0.041 |  | 0.340*** | 0.026 |  |
| λ | 0.160** | 0.063 | |  | 0.118** | 0.047 |  | 0.159*** | 0.011 |  |
| θ1 | 0.974*** | 0.121 | |  | 1.140*** | 0.078 |  | 0.773*** | 0.020 |  |
| θ2 | 1.578*** | 0.084 | |  | 2.105*** | 0.048 |  | 1.626*** | 0.021 |  |
| Observations | 7,100 |  | |  | 13,381 |  |  | 37,876 |  |  |

Notes: The Extended Estimating Equations (EEE) model estimates were reported in the table; Coef., coefficient; Adjusted Std.err. are standard errors adjusted for clustering at the patient level; UEBMI, Urban Employee-based Basic Medical Insurance scheme; URBMI, Urban Resident-based Basic Medical Insurance scheme; ICU, Intensive Care Unit; PCI, Percutaneous Coronary Intervention; ***, *P*<0.001, **, *P* <0.01, *, *P* <0.05.

**Table A3** The association between types of insurance and out-of-pocket expenses among different length of stay subgroups.

| **Associated Factors** | **Length of stay<15 Days** | | | **15≤Length of stay<30** | | | **Length of stay≥30** | | |
| --- | --- | --- | --- | --- | --- | --- | --- | --- | --- |
|  | Coef. | Adjusted Std.err. | Marginal Effect | Coef. | Adjusted Std.err. | Marginal Effect | Coef. | Adjusted Std.err. | Marginal Effect |
| **Gender** |  |  |  |  |  |  |  |  |  |
| Female (Reference) |  |  |  |  |  |  |  |  |  |
| Male | 0.167*** | 0.009 | 1,093.0 | 0.231*** | 0.018 | 2,092.9 | 0.245*** | 0.026 | 3,611.9 |
| **Age group** |  |  |  |  |  |  |  |  |  |
| 18≤Age<59 (Reference) |  |  |  |  |  |  |  |  |  |
| 60≤Age<69 | -0.125*** | 0.014 | -804.6 | -0.165*** | 0.037 | -1,462.0 | -0.223** | 0.097 | -3,180.7 |
| 70≤Age<79 | -0.165*** | 0.013 | -1,070.5 | -0.273*** | 0.034 | -2,430.5 | -0.431*** | 0.089 | -6,159.1 |
| ≥80 | -0.310*** | 0.014 | -1,886.5 | -0.402*** | 0.034 | -3,369.3 | -0.537*** | 0.089 | -7,130.6 |
| **Insurance type** |  |  |  |  |  |  |  |  |  |
| URBMI (Reference) |  |  |  |  |  |  |  |  |  |
| UEBMI | -0.493*** | 0.015 | -3,926.1 | -0.741*** | 0.029 | -8,605.8 | -0.917*** | 0.052 | -18,400.5 |
| **ICU admission** | 1.847*** | 0.412 | 29,284.5 | 1.267*** | 0.119 | 19,153.6 | 1.344*** | 0.175 | 34,590.1 |
| **PCI operation** | 1.738*** | 0.012 | 16,051.0 | 1.525*** | 0.032 | 18,781.9 | 1.235*** | 0.070 | 23,429.5 |
| **Hospital level** |  |  |  |  |  |  |  |  |  |
| Primary (Reference) |  |  |  |  |  |  |  |  |  |
| Secondary | 0.122*** | 0.021 | 843.2 | 0.193*** | 0.030 | 1,868.7 | 0.213*** | 0.039 | 3,383.0 |
| Tertiary | 0.814*** | 0.024 | 4,397.6 | 0.931*** | 0.033 | 7,119.9 | 1.017*** | 0.041 | 12,491.4 |
| **Year** |  |  |  |  |  |  |  |  |  |
| Year 2008 (Reference) |  |  |  |  |  |  |  |  |  |
| Year 2009 | 0.040** | 0.018 | 268.0 | 0.114*** | 0.033 | 1,070.7 | 0.216*** | 0.044 | 3,400.1 |
| Year 2010 | -0.002 | 0.019 | -13.2 | 0.077** | 0.032 | 713.8 | 0.129*** | 0.044 | 1,985.3 |
| Year 2011 | -0.322*** | 0.017 | -1,978.6 | -0.221*** | 0.029 | -1,927.2 | -0.054 | 0.039 | -791.4 |
| Year 2012 | -0.347*** | 0.016 | -2,148.9 | -0.218*** | 0.028 | -1,924.6 | -0.056 | 0.036 | -827.7 |
| **Comorbidity** |  |  |  |  |  |  |  |  |  |
| None (Reference) |  |  |  |  |  |  |  |  |  |
| Hypertension | -0.116*** | 0.012 | -744.0 | -0.150*** | 0.018 | -1,317.9 | -0.159*** | 0.026 | -2,264.4 |
| Diabetes mellitus | -0.009 | 0.019 | -59.6 | -0.099*** | 0.025 | -876.8 | -0.008 | 0.041 | -119.7 |
| Constant | -0.747*** | 0.032 |  | -0.127** | 0.056 |  | 0.587*** | 0.110 |  |
| λ | 0.093*** | 0.016 |  | 0.162*** | 0.026 |  | 0.118** | 0.046 |  |
| θ1 | 0.649*** | 0.020 |  | 0.923*** | 0.035 |  | 0.896*** | 0.051 |  |
| θ2 | 1.499*** | 0.033 |  | 1.613*** | 0.027 |  | 1.934*** | 0.065 |  |
| Observations | 39,037 |  |  | 13,274 |  |  | 6,046 |  |  |

Notes: The Extended Estimating Equations (EEE) model estimates were reported in the table; Coef., coefficient; Adjusted Std.err. are standard errors adjusted for clustering at the patient level; UEBMI, Urban Employee-based Basic Medical Insurance scheme; URBMI, Urban Resident-based Basic Medical Insurance scheme; ICU, Intensive Care Unit; PCI, Percutaneous Coronary Intervention; ***, *P*<0.001, **, *P* <0.01.
