# Supplementary material for: Direct medical costs of ischemic heart disease in urban Southern China: a 5-year retrospective analysis of an all-payer health claims database in Guangzhou City
Source: Front Public Health. 2023 May 9;11:1146914. doi: 10.3389/fpubh.2023.1146914 (PMC10203198; doi:10.3389/fpubh.2023.1146914)
Supplement: Supplementary file 1 [file Data_Sheet_1.PDF]

Dear editor and anonymous reviewers,

We are most grateful for your positive comments and constructive suggestions for revisions of our manuscript originally titled as “Direct medical costs of ischemic heart disease in urban Southern China: A 5-year retrospective analysis of an all-payer health claims database in Guangzhou City” (Manuscript ID: 1146914). We have revised the manuscript following your valuable suggestions. Detailed responses to your comments are listed below. The associated changes are highlighted in red in the revised manuscript.

We look forward to hearing from your decision when it is made. We would be glad to respond to any further questions and comments that you may have.

	**Reviewers’ comments**	**Authors’ responses**
	**Reviewer #1**	
1	- The improvement of the language of this manuscript is needed.	Thank you for this suggestion. As suggested, we have invited Professor Donglan Zhang from New York University Long Island School of Medicine to improve the language of this manuscript.
2	- The dependent variable was direct medical costs, why not the out-of-pocket expenditures. Out-of-pocket costs better reveal the disparities in health insurance schemes.	Thank you for this suggestion.<br>1) As suggested, we added a new Table “**Table 3 Ischemic heart disease patient characteristics associated with direct medical costs and out-of-pocket expenses**” to compare the differences in OOP expenses by types of insurance under different characteristics.<br>(Page 18; also attached at the end of this response letter)<br><br>2) One new paragraph presenting the findings of this new Table 3 was added in the **Results** section.<br>***Patient characteristics associated with direct medical costs and out-of-pocket expenses by type of insurance***<br>“Direct medical costs and OOP expenses for patients with IHD across different types of insurance significantly differed by gender, age, hospital level, LOS and comorbidities (all P<0.001) (see Table 3). For the overall samples, UEBMI subgroup and URBMI subgroup, male patients incurred higher direct medical costs and OOP expenses than female patients. Patients aged 60-69 years old had the highest direct medical costs for overall samples and two insurance subgroups. And patients aged 18-59 years old had the highest OOP expenses for overall samples and the UEBMI subgroup, but those aged 60-69 years old had the highest OOP expenses for the URBMI subgroup.

In addition, among the overall samples and all subgroups, the mean direct medical costs and OOP expenses of IHD patients recruited in tertiary hospitals were far more than those of patients staying in primary hospitals. Patients with LOS longer than 30 days had higher direct medical costs and OOP expenses than patients with shorter LOS. Patients having diabetes mellitus had higher direct medical cost than those with hypertension."

(Page 6, Lines 232-243)

3) Further, a new Table "**Table 4 Factors associated with direct medical costs and out-of-pocket expenses of ischemic heart disease (EEE model)**" has been added to explore the potential associated factors of out-of-pocket (OOP) expenses of IHD.

(Pages 19-20; also attached at the end of this response letter)

4) Some new paragraphs presenting the findings of this new Table 4 was added in the **Results** section.

Associated factors of direct medical costs and out-of-pocket expenses

"This study found that the types of insurance, PCI operation, gender, age, hospital levels, ICU admission, LOS, and comorbidities (hypertension and diabetes mellitus) were significantly associated with direct medical costs and OOP expenses of IHD. Compared with patients covered by the URBMI scheme, the direct medical costs for IHD patients insured by the UEBMI scheme were significantly higher (CNY 692.5) (USD 109.7), and their OOP expenses were significantly lower (CNY 5,416.1) (USD 858.0) (P<0.001), after controlling for other covariates (P<0.001).

Male patients had significantly higher direct medical costs and OOP expenses than their female counterparts (P<0.001). Compared with patients aged 18-59, patients 60-69 and 70-79 had CNY 918.6 (USD 145.5) and CNY 1,460.3 (USD 231.3) lower OOP expenses (P<0.001), and patients aged 80 and above had CNY 1,159.3 (USD 183.7) lower direct medical costs and CNY 2,349.4 (USD 372.2) lower OOP expenses (P<0.001). Patients having PCI operation and ICU admission incurred significantly higher direct medical costs and OOP expenses (P<0.001). Compared to patients treated in primary hospitals, IHD patients staying in secondary hospitals had CNY 7,093.3 (USD 1,123.7) higher direct medical costs and CNY 1,192.9 (USD 189.0) higher OOP expenses (P<0.001), and patients staying in tertiary hospitals had CNY 19,006.6 (USD 3,010.9)

		higher direct medical costs and CNY 5,505.5 (USD 872.2) higher OOP expenses (*P*<0.001). In comparison with LOS of less than 15 days, patients with the LOS between 15 and 30 days had CNY16,432.5 (USD 2,603.2) higher direct medical costs and CNY 3,705.0 (USD 586.9) higher OOP expenses (*P*<0.001), and patients with the LOS of more than 30 days had CNY53,216.7 (USD 8,430.4) higher direct medical costs and CNY 10,763.1 (USD 1,705.0) higher OOP expenses(*P*<0.001). Also, IHD patients with hypertension incurred lower direct medical costs and lower OOP expenses (*P*<0.001), but patients with diabetes mellitus had higher direct medical costs and lower OOP expenses (*P*<0.001)."<br>(Pages 6-7, Lines 248-269)<br><br>5) We also added a few sentences to explain the differences in OOP expenses between two insurance schemes in the **Discussion** section:<br>***Difference in costs between two insurance schemes***<br>"The regression findings also suggested that UEBMI enrollees had significantly higher direct medical costs with IHD including both inpatient and outpatient care, but had lower OOP expenses than URBMI enrollees."<br>(Page 9, Lines 360-363)<br><br>6) Additionally, a new Figure "**Figure 3** Time trends of out-of-pocket expenses of ischemic heart disease patients." has been added to explore the time trends of out-of-pocket (OOP) expenses of IHD.<br>(Page 26, also attached at the end of this response letter.)<br><br>7) Some new paragraphs presenting the findings of this new **Figure 3** was added in the **Results** section.<br>***Time trends of direct medical costs and out-of-pocket expenses by insurance type***<br>"As shown in Figure 2, the direct medical costs for all patients with IHD increased firstly from CNY 24,736.0 (USD 3,918.6) in 2008 to CNY 28,282.0 (USD 4,480.3) in 2009. However, there was a turning point in 2009 and then the direct medical costs decreased 1.9% per year during the period of 2009-2012. The time trends of OOP expenses were similar to the direct medical costs. The OOP expenses of all IHD patients increased from CNY 9,351.1 (USD 1,481.4) in 2008 to CNY 10,840.9 (USD 1,717.4) in 2009 (see Figure 3) and there was also a turning point in 2009. The OOP expenses of all sample decreased during the period of 2009-2012.<br>When comparing two health insurance schemes, the direct

		medical costs for UEBMI patients initially increased from CNY 24,967.9 (USD 3,955.3) in 2008 to CNY 29,070.9 (USD 4,605.3) in 2009 and then decreased 2.1% per year during the period of 2009-2012. On the contrary, the direct medical costs of IHD for URBMI patients increased 3.0% per year from CNY 19,325.8 (USD 3,061.5) in 2008 to CNY 21,730.9 (USD 3,442.5) in 2012. The time trends of OOP expenses among UEBMI subgroup and URBMI subgroup were similar."<br>(Page 6, Lines 214-226)
3	- In lines 29-32, the temporal trends in costs under both insurances should be clearly indicated, such as increasing or decreasing between 2008-2012, and the degree. You have learned those factors were associated with medical costs, just briefly point out which factors contribute to the increase or decrease in medical costs in the abstract.	1) Thank you for this suggestion. We have added detailed explanation of the temporal trends in costs under both insurances in the **Abstract** section:<br><br>"The direct medical costs and OOP expenses for all patients increased from 2008 to 2009, and then decreased during the period of 2009-2012. The time trends of direct medical costs between the UEBMI and URBMI patients were different during the period of 2008-2012."<br>(Page 1, Lines 28-31).<br><br>2) As suggested, we have added detailed explanation of the factors associated with medical costs in the **Abstract** section:<br>"The regression analysis indicated that the UEBMI enrollees had higher direct medical costs (P<0.001) but had lower OOP expenses (P<0.001) than the URBMI enrollees. Male patients, patients having percutaneous coronary intervention operation and intensive care unit admission, patients treated in secondary hospitals and tertiary hospitals, patients with the LOS of 15-30 days, 30 days and longer had significantly higher direct medical costs and OOP expenses (all P<0.001)."<br>(Pages 1-2, Lines 31-35).
4	- In line 34, "It was important to" is not academic language. In lines 34-37, the conclusion should briefly summarize the key points derived from the current study, and the sentence can be removed.	1) Thank you for this suggestion. We have deleted this sentence and revised the conclusion in the **Abstract** section:<br>"The direct medical costs and OOP expenses for patients with IHD in China were found to be high and varied between two medical insurance schemes. The type of insurance was significantly associated with direct medical costs and OOP expenses of IHD."<br>(Page 2, Lines 36-38).<br><br>2) Accordingly, we have also revised the conclusion in the **Conclusion** section:<br>"The direct medical costs and OOP expenses of patients

		with IHD for Chinese urban population were high and different across two basic health insurance schemes. Types of health insurance, gender, age, hospital levels, LOS, ICU admission, PCI operation and comorbidities were significantly associated with the direct medical costs and OOP expenses of IHD. Therefore, policymakers should aim to reduce disparities across different health insurance schemes in terms of benefit packages and narrow the economic burden gap among IHD patients covered by different insurance schemes."<br>(Page 10, Lines 432-437).
5	- Why were patients aged under 18 years excluded?	Thanks for your question. The reason why we excluded patients aged under 18 years was provided below:<br>Firstly, we noticed that IHD is most common in people over 45 years old (Locatelli, 2023), and there are very few cases of IHD under the age of 18. And we found that there were only two individuals under the age of 18 in our dataset before sample selection.<br>Secondly, the UEBMI enrollees were all aged 18 years and above. To ensure the comparability of the two insurance schemes, we excluded a very small number of individuals under 18 years of age.<br>Furthermore, we found that previous literature(Y. Wang et al., 2022; Yu, Zhang, Yao, & Cao, 2020; Zhao et al., 2014) comparing the two insurance schemes also excluded samples under 18 years of age. Therefore, patients under 18 years of age were excluded in this study.
6	- Lines 146-152 are redundant	Thank you for this suggestion. We have deleted lines 146-152 of the original manuscript.
7	- Tobit model was conducted in many studies of factors associated with medical costs, and the EEE model was used in the current study. What's your consideration?	Thanks for your question. The reason why we choose the EEE model was provided below:<br>1) Healthcare cost data usually have a skewed distribution, and such problems were often dealt with by logarithmic transformation of the cost data. The dominant approach to modelling health care costs in the recent literature has been the use of GLM. Advantages of the GLM approach are that predictions are made on the raw cost scale, so that no retransformation is required, and that they allow for heteroskedasticity through the choice of distributional family, albeit limited to specifications of the conditional variance that are prespecified functions of the mean (Jones, 2010). In response to the difficulties of selecting the

		appropriate link function and distribution by the traditional GLM model, the extension of GLM -- the extended estimating equations (EEE) approach was used in this study (Basu & Rathouz, 2005). In contrast to the difficulties of selecting the appropriate link function and distribution by the traditional GLM model, the EEE model allows for estimation of flexible link and variance functions using the data at hand, thereby reducing bias and inefficiency in estimation (Basu & Rathouz, 2005).<br><br>2) Meanwhile, we learned that Tobit model is suitable for studying variables with the following characteristics: The variable has a lower, or upper, limit and takes on the limiting value for a substantial number of respondents. For the remaining respondents, the variable takes on a wide range of values above, or below, the limit (Tobin, 1958). Tobit model was usually used in left-censored data with a value of zero (Al-Hanawi, Mwale, & Qattan, 2021; Hyun & Kan, 2022; Peng et al., 2021) or variables with an upper limit (Li, Hung, Shi, Fu, & Qian, 2023). In our sample, there were no zero values for the direct medical costs and out-of-pocket expenses due to our insurance reimbursement claims dataset. Therefore, the Tobit model is not suitable for our sample.<br><br>3) Furthermore, we have tried Tobit model and the detailed result tables "**Appendix Table 1:** Factors associated with direct medical costs and out-of-pocket expenses of ischemic heart disease (Tobit model)" were presented at the end of this response file as the appendix.<br>We found that the regression results of Tobit model were similar as the results of EEE model in our manuscript. Therefore, we have decided to use EEE model in our manuscript.
8	- In lines 359 to 365, I think the explanation is not plausible	Indeed, some previous studies have found that older patients with IHD incurred higher medical costs than younger patients (Ding et al., 2017; Schlatter et al., 2017). However, our finding was similar to another study in China (Q. Wang, Zhang, Wang, Zhang, & Zhang, 2019), which also found that younger patients aged 60 years and below had significantly higher hospitalization costs than those aged 80 years above. The possible reason may be the sample selection problem. Therefore, we revised the explanation in **Discussion** section. Please see more details in the revised manuscript.<br><br>***Associated factors of direct medical costs and Out-of-Pocket***

		Expenses<br><br>"Age. In this study, we demonstrated that those aged 80 and above had significantly lower direct medical costs and lower OOP expenses than those aged under 60. Previous studies found that older patients with IHD incurred higher medical costs than younger patients(Ding et al., 2017; Schlatter et al., 2017). However, our finding was similar to another China-based study (Q. Wang et al., 2019),which also found that younger patients aged 60 years and below had significantly higher hospitalization costs than those aged 80 years above. This may be due to the variation in sample selection among different studies."<br>(Page 9, Lines 387-392)
9	- The discussion section is a little lengthy, please address the significant and particular findings of this research and refine your discussion, especially the associated factors.	Thank you for this suggestion. As suggested, we cut down the discussion of association between age, gender and direct medical costs in the **Discussion** section. Please see more details in the revised manuscript.<br><br>***Associated factors of direct medical costs and Out-of-Pocket Expenses***<br><br>"Age. In this study, we demonstrated that those aged 80 and above had significantly lower direct medical costs and lower OOP expenses than those aged under 60. Previous studies found that older patients with IHD incurred higher medical costs than younger patients(Ding et al., 2017; Schlatter et al., 2017). However, our finding was similar to another China-based study (Q. Wang et al., 2019),which also found that younger patients aged 60 years and below had significantly higher hospitalization costs than those aged 80 years above. This may be due to the variation in sample selection among different studies.<br><br>Gender. In consistent with previous studies (Ding et al., 2017; Schlatter et al., 2017; Walker et al., 2016; Q. Wang et al., 2019), we found that male patients had higher direct medical costs than female patients. The possible reasons might be the higher IHD burden (Zhang et al., 2018), higher morbidity of IHD (Wei et al., 2022), and more consumption of drinking and smoking (Zhou et al., 2016) for male patients than female patients, and thus male patients may suffer from more serious cardiovascular disease and incurred higher expenditures."<br><br>(Pages 9-10, Lines 387-397)
10	- Please check the language	Thank you for this suggestion. We checked the language and expression, and revised the manuscript.

	and expression as follows:<br>Line 15: "different type"<br>Lines 24-25: the "average" should be added.<br>Lines 177-178.<br>Line 299: A dot is absent.	1) As suggested, we changed "different type" to "types" in the **Abstract** section.<br>(Page 1, Line 16, Line 22).<br><br>2) We added an "average" in Line 27, Line 31 in the **Abstract** section.<br>(Page 1, Line 24, Line 26).<br><br>3) We deleted "There were" in the **Results** section.<br>(Page 5, Line 179).<br><br>4) We added a dot in the **Discussion** section.<br>(Page 8, Line 329).
11	- Please check the format of your references against the latest articles in the journal.	Thank you for this suggestion. We checked the format of our references, and revised the references in our manuscript. Please refer to the revised manuscript for details in the **References** section.<br>(Pages 12-15, Lines 482-668).
	**Reviewer #2**	
	This is a valuable empirical study on the economic burden of disease, its salient feature is the large sample collected from medical insurance claims database, including both inpatient costs and outpatient costs, real world data and robust analysis. Some suggestions as follows.	We appreciate the reviewer's thorough assessment of our manuscript.
1	- Focus on the core issues in the drivers for direct medical costs, eliminate insurance differences and the time trends. Suggest comparison the insurance system by heterogeneity test within regression analysis, it is easy to understand.	1) As suggested, we conducted a heterogeneity test to compare the insurance system, and the detailed result table "**New Table A1:** The association between types of insurance and direct medical costs among different hospital level subgroups." was presented at the end of this response file.<br>(Pages 21-22; also attached at the end of this response letter.)<br><br>2) Considering **Reviewer#1 Comment#2,** we added two new Tables "**New Table A2:** The association between types of insurance and out-of-pocket expenses among different hospital

		level subgroups." and "**New Table A3:** The association between types of insurance and out-of-pocket expenses among different length of stay subgroups." to compare the differences in the association between types of insurance and out-of-pocket expenses among different subgroups. The detailed result tables were presented at the end of this response file.<br>(Pages 23-26; also attached at the end of this response letter.)<br><br>3) One new paragraph was added in the **Method** section.<br><br>***Statistical analysis***<br><br>"Additionally, the heterogeneity analysis was used to examine the differences in the association between types of insurance and direct medical costs as well as OOP expenses among different subgroups."<br>(Pages 4-5, Lines 167-169)<br><br>4) One new paragraph presenting the findings of **New Table A1, New Table A2** and **New Table A3** was added in the **Results** section.<br><br>***Associated factors of direct medical costs and out-of-pocket expenses***<br><br>"The results of the heterogeneity analysis indicated that hospital level had an effect on the association between types of insurance and direct medical costs, while hospital level and LOS had an effect on the association between types of insurance and OOP expenses. Details on the results of heterogeneity analysis in this study are shown in Appendix A."<br>(Page 7, Lines 272-275)
2	- The results here are correlation factors rather than determinants of the economic burden of disease. The variables included here are mainly characteristic data of medical service utilization, such as hospital level and length of stay, etc. These variables are symbiotic correlation without causality. Individual factors and disease factors, which are more	1) Thank you for this suggestion. We agree with the reviewer that the results of our study are correlation factors rather than determinants of the economic burden of disease. As suggested, we changed "determinants/drivers" to "associated factors" in the revised manuscript.<br>(Page 1, Line 16, Line 17, Line 23;<br>Page 2, Line 38;<br>Page 3, Line 97;<br>Page 4, Line 133, Line 163;<br>Page 6, Line 247;<br>Page 8, Line 337;<br>Page 9, Line 386;<br>Page 19, Line 752;

	critical to determine the economic burden, are not introduced in this paper, so it is inaccurate to use the word-- "drivers". It is recommended to specify this setting in the study, or indicate this in limitation.	Page 21, Line 785;<br>Page 23, Line 801:<br>Page 25, Line 818).<br><br>2) Ideally, the individual factors and disease factors should be considered in the study. However, different from household survey data, detailed individual factors and disease factors (such as disease severity and duration), which are more critical to determine the economic burden, were not collected in the health insurance claims dataset. Therefore, we chose two dummy variables as proxy measures for the severity of disease – whether having an ICU admission, and having the operation of PCI in our analysis.<br><br>3) We have included this point in the **Limitations** section:<br>"Fourth, individual factors and disease factors, which were more critical to determine the economic burden, were not introduced in this study because such data were not available in the claims dataset."<br>(Page 10, Lines 426-428).
3	- Meanwhile, this study is more like an analysis of factors associated with the economic burden of disease. Patients with IHD here are only the samples, the specificity of IHD is not reflected in the analysis, such as case characteristics and treatment methods.	We agree with the reviewer that ideally the specificity of IHD such as case characteristics and treatment methods should be considered in our analysis. However, detailed case characteristics and treatment methods of IHD were not available in our insurance claims dataset.<br><br>We have mentioned this in the "**Limitations**" section:<br>"Finally, the specificity of IHD, such as case characteristics and treatment methods, was not reflected in our analysis due to the unavailability of such data in the claims dataset. Further studies could consider adding case characteristics and treatment methods to examine the determinants of IHD costs."<br>(Page 10, Lines 428-430).
4	- Such as the cardiac rehabilitation program in community health services center, it not mentioned in the context and discussion and pops up in suggestions. very abrupt.	We agree with the reviewer that suggestions about cardiac rehabilitation program were not appropriate. We have not mentioned the cardiac rehabilitation program in community health services center in the context and discussion. Therefore, we have deleted this from the "**Discussion"** section.
5	- The database 2008 to 2012 is a bit old, and it would	Thank you for this suggestion. It would be ideal if we can get the latest dataset to investigate the IHD costs. However, we can only

	be nice if it could be updated	access insurance claims data before 2012, due to administrative restrictions on data availability.<br><br>We have acknowledged this in the "**Limitations**" section：<br>"Third, the dataset was a little bit old due to administrative restrictions on data availability, which may not reveal the IHD costs and differences at present. Future studies could consider using a more recent claims dataset, as well as including people covered by all types of insurance, and examining the indirect costs for a more comprehensive evaluation of IHD costs."<br>(Page 10, Lines 422-426)
6	- In writing, mixed use of RMB and USD units.	Thank you for this suggestion. We have unified the unit as CNY(USD) and showed both RMB and USD for all expenditures. Please refer to the revised manuscript for details.
7	- insurance type shouldn't appear on the vertical axis of Table 1.	Thank you for this suggestion. As suggested, we have deleted insurance type on the vertical axis of Table 1. Please refer to the new Table1 in our revised manuscript.<br>(Page 16; also attached at the end of this response letter)
	**Editor**	
1	- We noticed the use of the phrase "elderly" in your manuscript. Please be advised that this is not correct terminology. Please replace all instances of this phrase with the phrase 'older adults'.	Thank you for this suggestion. We have revised the discussion of the association between age and direct medical costs, and there is no such word in the revised manuscript.<br><br>***Associated factors of direct medical costs and Out-of-Pocket Expenses***<br><br>"Age. In this study, we demonstrated that those aged 80 and above had significantly lower direct medical costs and lower OOP expenses than those aged under 60. Previous studies found that older patients with IHD incurred higher medical costs than younger patients(Ding et al., 2017; Schlatter et al., 2017). However, our finding was similar to another China-based study (Q. Wang et al., 2019),which also found that younger patients aged 60 years and below had significantly higher hospitalization costs than those aged 80 years above. This may be due to the variation in sample selection among different studies."<br><br>(Page 9, Lines 387-392)

New Table 1 Characteristics of patients with ischemic heart disease

Variables	Overall	UEBMI	URBMI
**No. Patients**	58,357	53,014	5,343
**Gender**			
Female	30,078 (51.5)	26,608 (50.2)	3,470 (64.9)
Male	28,279 (48.5)	26,406 (49.8)	1,873 (35.1)
**Age (years)**			
Mean±SD	72.0±11.4	71.8±11.4	74.1±11.0
Median（25th-75th）	74.0 (64.0-80.0)	74.0 (64.0-80.0)	75.0 (67.0-82.0)
**Age group**			
18≤Age<59	9,129 (15.6)	8,563 (16.2)	566 (10.6)
60≤Age<69	12,016 (20.6)	10,912 (20.6)	1,104 (20.7)
70≤Age<79	21,326 (36.5)	19,409 (36.6)	1,917 (35.9)
≥80	15,886 (27.2)	14,130 (26.7)	1,756 (32.9)
**Hospital level**			
Primary	7,100 (12.2)	6,220 (11.7)	880 (16.5)
Secondary	13,381 (22.9)	11,917 (22.5)	1,464 (27.4)
Tertiary	37,876 (64.9)	34,877 (65.8)	2,999 (56.1)
**ICU admission**	80 (0.1)	73 (0.1)	7 (0.1)
**PCI operation**	9,846 (16.9)	9,240 (17.4)	606 (11.3)
**Length of stay(days)**			
Mean±SD	16.1±21.5	16.2±22.0	14.3±16.2
Median（25th-75th）	11.0 (7.0-17.0)	11.0 (7.0-17.0)	10.0 (7.0-16.0)
Days＜15	39,037 (66.9)	35,285 (66.6)	3,752 (70.2)
15≤Days＜30	13,274 (22.7)	12,128 (22.9)	1,146 (21.4)
Days≥30	6,046 (10.4)	5,601 (10.6)	445 (8.3)
**Admission year**			
Year 2008	7,832 (13.4)	7,510 (14.2)	322 (6.0)
Year 2009	9,792 (16.8)	8,835 (16.7)	957 (17.9)
Year 2010	9,547 (16.4)	8,671 (16.4)	876 (16.4)
Year 2011	13,945 (23.9)	12,616 (23.8)	1,329 (24.9)
Year 2012	17,241 (29.5)	15,382 (29.0)	1,859 (34.8)
**Comorbidities**			
None	42,024 (72.0)	38,096 (71.9)	3,928 (73.5)
Hypertension	13,544 (23.2)	12,332 (23.3)	1,212 (22.7)
Diabetes mellitus	4,177 (7.2)	3,870 (7.3)	307 (5.7)

Notes: n (%) for categorical variables and mean± standard deviation (SD) or median (25-27th) for continuous variables; URBMI, Urban Resident-based Basic Medical Insurance scheme; UEBMI, Urban Employee-based Basic Medical Insurance scheme; ICU, Intensive Care Unit; PCI, Percutaneous Coronary Intervention

New Table 3 Ischemic heart disease patient characteristics associated with direct medical costs and out-of-pocket expenses

**Patient Characteristics**	Direct Medical Costs							Out-of-Pocket Expenses						
	**Overall**		**UEBMI**		**URBMI**			**Overall**		**UEBMI**		**URBMI**		
**No. Patients**	**n=58,357**		**n=53,014**		**n=5,343**		***P*-value**	**n=58,357**		**n=53,014**		**n=5,343**		***P*-value**
	Mean	SD	Mean	SD	Mean	SD		Mean	SD	Mean	SD	Mean	SD	
**Gender**							*P*<0.001							*P*<0.001
Female	22,234.1	28,941.0	22,739.7	29,382.2	18,357.6	24,968.8		6,402.3	9.988.05	6,017.8	9,201.7	9,348.6	14,342.9	
Male	32,350.5	40,037.1	32,796.7	40,474.9	26,060.0	32,622.3		10,183.9	14,299.6	9,919.3	13,845.1	13,913.6	19,237.7	
**Age group**							*P*<0.001							*P*<0.001
18≤Age<59	29,574.7	36,748.8	29,949.3	37,048.8	23,907.0	31,354.0		10,981.2	14,937.5	10,864.1	14,691.5	12,752.2	18,182.6	
60≤Age<69	30,148.6	39,034.3	30,634.5	39,710.2	25,346.3	31,195.4		9,729.9	13,684.7	9,305.2	13,041.7	13,927.7	18,384.8	
70≤Age<79	28,286.4	35,902.4	28,880.3	36,445.3	22,274.0	29,189.7		8,348.0	12,301.2	8,007.5	11,669.4	11,795.7	17,080.7	
≥80	21,912.8	28,898.3	22,633.3	29,510.2	16,115.1	22,584.3		5,373.2	8,876.0	5,100.2	8,250.7	7,570.3	12,634.1	
**Hospital level**							*P*<0.001							*P*<0.001
Primary	7,804.5	10,128.6	7,904.0	9,986.9	7,206.1	10,929.1		1,409.2	2,391.8	1,280.2	1,829.2	2,184.5	4,395.3	
Secondary	12,258.0	17,608.3	12,516.3	18,135.6	10,182.6	12,405.7		2,724.9	4,109.2	2,563.0	3,956.6	4,026.1	4,988.3	
Tertiary	35,034.0	39,015.7	35,453.2	39,448.8	30,053.7	33,044.7		11,111.2	14,041.6	10,644.1	13,389.1	16,661.4	19,405.9	
**Length of stay(days)**							*P*<0.001							*P*<0.001
Days<15	20,118.4	25,935.9	20,611.8	26,357.3	15,478.5	21,017.5		6,934.9	10,559.3	6,773.3	10,284.8	8,454.5	12,758.8	
15≤Days<30	34,271.3	36,390.3	34,803.2	36,808.4	28,642.6	31,089.8		10,013.1	13,855.7	9,579.7	13,220.4	14,600.0	18,742.2	
Days≥30	56,784.1	57,898.8	57,437.1	58,688.0	48,564.6	46,109.8		12,722.5	17,545.4	11,939.5	16,334.0	22,577.5	26,853.6	
**Comorbidity**							*P*<0.001							*P*<0.001
None	28,313.4	36,226.2	28,984.1	36,844.0	21,808.4	28,777.0		8,940.1	13,018.5	8,674.4	12,507.4	11,516.8	16,992.9	
Hypertension	22,411.2	30,020.8	22,833.8	30,432.4	18,111.9	25,066.3		5,861.7	9,718.8	5,570.2	9,196.5	8,827.3	13,619.0	
Diabetes mellitus	30,953.5	38,478.9	31,637.9	39,055.6	22,325.0	28,973.4		8,232.6	12,425.0	7,997.0	12,027.2	11,203.0	16,380.0	

Notes: P-values are based on the Friedman's two-way non-parametric analysis of variance test; All costs were based on a constant 2012 Chinese Yuan (CNY); UEBMI, Urban Employee-based Basic Medical Insurance scheme; URBMI, Urban Resident-based Basic Medical Insurance scheme; SD, standard deviation; ICU, Intensive Care Unit; PCI, Percutaneous Coronary Intervention

New Table 4 Factors associated with direct medical costs and out-of-pocket expenses of ischemic heart disease (EEE model)

Associated Factors	Direct Medical Costs			Out-of-Pocket Expenses		
	n=58,357			n=58,357		
	Coef.	Adjusted Std.err.	Marginal Effect	Coef.	Adjusted Std.err.	Marginal Effect
**Gender**						
Female (Reference)						
Male	0.118***	0.006	2,962.8	0.191***	0.008	1,464.7
**Age group**						
18≤Age<59(Reference)						
60≤Age<69	0.001	0.011	3.4	-0.122***	0.013	-918.6
70≤Age<79	-0.013	0.010	-327.0	-0.192***	0.012	-1,460.3
≥80	-0.047***	0.010	-1,159.3	-0.326***	0.013	-2,349.4
**Insurance type**						
URBMI(Reference)						
UEBMI	0.028***	0.009	692.5	-0.577***	0.014	5,416.1
**Hospital level**						
Primary (Reference)						
Secondary	0.265***	0.008	7,093.3	0.148***	0.015	1,192.9
Tertiary	0.870***	0.011	19,006.6	0.842***	0.016	5,505.5
**ICU admission**	0.584***	0.008	76,882.9	1.500***	0.105	21,401.4
**PCI operation**	1.432***	0.013	51,211.0	1.665***	0.012	17,893.0
**Length of stay(days)**						
Days<15 (Reference)						
15≤Days<30	1.707***	0.087	16,432.5	0.436***	0.010	3,705.0
Days≥30	1.509***	0.009	53,216.7	1.005***	0.015	10,763,1
**Year**						
Year 2008 (Reference)						
Year 2009	0.112***	0.011	2,888.0	0.087***	0.015	688.4
Year 2010	0.128***	0.011	3,321.7	0.043***	0.016	339.1
Year 2011	0.151***	0.010	3,898.9	-0.230***	0.014	-1,694.9
Year 2012	0.152***	0.010	3,908.1	-0.240***	0.013	-1,777.0
**Comorbidity**						
None (Reference)						

Hypertension	-0.073***	0.006	-1,806.4	-0.127***	0.009	-951.4
Diabetes mellitus	0.045***	0.010	1,139.8	-0.043***	0.014	-329.4
λ	0.265***	0.009		0.160***	0.010	
θ1	0.575***	0.011		0.819***	0.019	
θ2	1.829***	0.018		1.654***	0.031	

Notes: The Extended Estimating Equations (EEE) model estimates were reported in the table; Coef., coefficient; Adjusted Std.err. are standard errors adjusted for clustering at the patient level; UEBMI, Urban Employee-based Basic Medical Insurance scheme; URBMI, Urban Resident-based Basic Medical Insurance scheme; ICU, Intensive Care Unit; PCI, Percutaneous Coronary Intervention; ***, *P*<0.001, **, *P* <0.01, *, *P* <0.05.

Appendix A

New Table A1 The association between types of insurance and direct medical costs among different hospital level subgroups.

Associated Factors	Primary			Secondary			Tertiary		
	Coef.	Adjusted Std.err.	Marginal Effect	Coef.	Adjusted Std.err.	Marginal Effect	Coef.	Adjusted Std.err.	Marginal Effect
**Gender**									
Female(Reference)									
Male	0.056***	0.015	1141.5	0.125***	0.013	2557.059	0.135***	0.008	3625.8
**Age group**									
18≤Age<59 (Reference)	0.056**	0.025	1146.5	0.017	0.026	354.0719	-0.001	0.012	-26.7
60≤Age<69	0.027	0.022	548.7	0.02	0.024	417.511	-0.013	0.011	-343.9
70≤Age<79	-0.01	0.02	-210.8	-0.029	0.023	-591.078	-0.049***	0.012	-1303.7
≥80	0.056**	0.025	1146.5	0.017	0.026	354.0719	-0.001	0.012	-26.7
**Insurance type**									
URBMI(Reference)									
UEBMI	0.107***	0.019	2113.0	0.084***	0.016	1678.746	0.007	0.013	196.1
**ICU admission**	2.857***	0.675	109800.5	2.331***	0.359	110563.2	2.339***	0.156	90036.5
**PCI operation**	2.075***	0.045	61000.0	1.944***	0.031	60557.95	1.684***	0.015	55308.6
**Length of stay(days)**									
Days<15 (Reference)									
15≤Days<30	0.331***	0.033	7146.0	0.536***	0.027	12303.25	0.614***	0.011	17579.4
Days≥30	1.037***	0.061	26739.1	1.349***	0.038	40984.79	1.636***	0.03	55019.8
**Year**									
Year 2008 (Reference)									
Year 2009	0.107***	0.026	2236.4	0.115***	0.02	2439.277	0.098***	0.014	2662.9
Year 2010	0.125***	0.027	2615.1	0.144***	0.02	3074.624	0.098***	0.014	2643.2

Year 2011	0.154***	0.028	3217.1	0.245***	0.021	5290.845	0.098***	0.013	2644.1
Year 2012	0.219***	0.031	4584.1	0.255***	0.021	5469.884	0.091***	0.012	2459.6
**Comorbidity**									
None (Reference)									
Hypertension	-0.031**	0.014	-628.3	-0.092***	0.012	-1849.84	-0.079***	0.009	-2082.6
Diabetes mellitus	0.011	0.024	231.2	0.046**	0.019	963.9842	0.046***	0.014	1231.0
Constant	-1.311***	0.083		-1.465***	0.053		-0.700***	0.02	
	0.528***	0.072		0.260***	0.039		0.594***	0.021	
	0.593***	0.047		0.647***	0.032		0.589***	0.013	
	1.556***	0.075		1.884***	0.046		1.460***	0.025	
Observations	7,100			13,381			37,876		

Notes: The Extended Estimating Equations (EEE) model estimates were reported in the table; Coef., coefficient; Adjusted Std.err. are standard errors adjusted for clustering at the patient level; UEBMI, Urban Employee-based Basic Medical Insurance scheme; URBMI, Urban Resident-based Basic Medical Insurance scheme; ICU, Intensive Care Unit; PCI, Percutaneous Coronary Intervention; ***, P<0.001, **, P <0.01, *, P <0.05.

New Table A2 The association between types of insurance and out-of-pocket expenses among different hospital level subgroups.

Associated Factors	Primary			Secondary			Tertiary		
	Coef.	Adjusted Std.err.	Marginal Effect	Coef.	Adjusted Std.err.	Marginal Effect	Coef.	Adjusted Std.err.	Marginal Effect
**Gender**									
Female(Reference)									
Male	0.177***	0.031	934.1	0.174***	0.018	1011.8	0.197***	0.009	1742.4
**Age group**									
18≤Age<59 (Reference)									
60≤Age<69	0.038	0.05	206.9	-0.083**	0.037	-474.6	-0.150***	0.015	-1288.7
70≤Age<79	-0.034	0.044	-180.6	-0.135***	0.034	-780.7	-0.221***	0.014	-1930.7
≥80	-0.156***	0.044	-803.4	-0.273***	0.036	-1497.1	-0.345***	0.016	-2867.1
**Insurance type**									
URBMI(Reference)									
UEBMI	-0.288***	0.049	-1704.3	-0.462***	0.034	-3204.6	-0.696***	0.017	-7779.6
**ICU admission**	2.405***	0.322	35988.9	2.115***	0.273	32627.3	1.375***	0.106	21300.9
**PCI operation**	2.363***	0.052	20383.1	2.159***	0.036	19772.9	1.585***	0.01	19993.7
**Length of stay(days)**									
Days<15 (Reference)									
15≤Days<30	0.331***	0.04	1906.1	0.433***	0.034	2811.6	0.453***	0.012	4445.2
Days≥30	0.892***	0.083	6426.9	1.007***	0.054	8419.0	1.043***	0.022	12905.8
**Year**									
Year 2008 (Reference)									
Year 2009	0.144***	0.056	801.8	0.098***	0.029	589.4	0.073***	0.019	658.8
Year 2010	0.076	0.053	414.4	0.044	0.028	262.6	0.032	0.019	282.4
Year 2011	-0.048	0.05	-254.2	-0.013	0.023	-77.3	-0.344***	0.017	-2846.7
Year 2012	0.022	0.048	120.2	-0.045**	0.021	-261.0	-0.363***	0.016	-3032.3

**Comorbidity**									
None (Reference)									
Hypertension	-0.068**	0.028	-357.7	-0.159***	0.018	-890.5	-0.131***	0.012	-1132.2
Diabetes mellitus	-0.088*	0.048	-452.9	-0.055**	0.026	-315.5	-0.032*	0.018	-281.6
Constant	-1.276***	0.088		-0.855***	0.041		0.340***	0.026	
	0.160**	0.063		0.118**	0.047		0.159***	0.011	
	0.974***	0.121		1.140***	0.078		0.773***	0.02	
	1.578***	0.084		2.105***	0.048		1.626***	0.021	
Observations	7,100			13,381			37,876		

Notes: The Extended Estimating Equations (EEE) model estimates were reported in the table; Coef., coefficient; Adjusted Std.err. are standard errors adjusted for clustering at the patient level; UEBMI, Urban Employee-based Basic Medical Insurance scheme; URBMI, Urban Resident-based Basic Medical Insurance scheme; ICU, Intensive Care Unit; PCI, Percutaneous Coronary Intervention; ***, P<0.001, **, P <0.01, *, P <0.05.

New Table A3 The association between types of insurance and out-of-pocket expenses among different length of stay subgroups.

Associated Factors	LOS<15 Days			15≤LOS<30			LOS≥30		
	Coef.	Adjusted Std.err.	Marginal Effect	Coef.	Adjusted Std.err.	Marginal Effect	Coef.	Adjusted Std.err.	Marginal Effect
**Gender**									
Female(Reference)									
Male	0.167***	0.009	1093.0	0.231***	0.018	2092.891	0.245***	0.026	3611.9
**Age group**									
18≤Age<59 (Reference)									
60≤Age<69	-0.125***	0.014	-804.6	-0.165***	0.037	-1462.004	-0.223**	0.097	-3180.7
70≤Age<79	-0.165***	0.013	-1070.5	-0.273***	0.034	-2430.515	-0.431***	0.089	-6159.1
≥80	-0.310***	0.014	-1886.5	-0.402***	0.034	-3369.313	-0.537***	0.089	-7130.6
**Insurance type**									
URBMI(Reference)									
UEBMI	-0.493***	0.015	-3926.1	-0.741***	0.029	-8605.279	-0.917***	0.052	-18400.5
**ICU admission**	1.847***	0.412	29284.5	1.267***	0.119	19153.6	1.344***	0.175	34590.1
**PCI operation**	1.738***	0.012	16051.0	1.525***	0.032	18781.94	1.235***	0.07	23429.5
**Hospital level**									
Primary (Reference)									
Secondary	0.122***	0.021	843.2	0.193***	0.03	1868.667	0.213***	0.039	3383.0
Tertiary	0.814***	0.024	4397.6	0.931***	0.033	7119.922	1.017***	0.041	12491.4
**Year**									
Year 2008 (Reference)									
Year 2009	0.040**	0.018	268.0	0.114***	0.033	1070.74	0.216***	0.044	3400.1
Year 2010	-0.002	0.019	-13.2	0.077**	0.032	713.7917	0.129***	0.044	1985.3
Year 2011	-0.322***	0.017	-1978.6	-0.221***	0.029	-1927.224	-0.054	0.039	-791.4
Year 2012	-0.347***	0.016	-2148.9	-0.218***	0.028	-1924.551	-0.056	0.036	-827.7

**Comorbidity**									
None (Reference)									
Hypertension	-0.116***	0.012	-744.0	-0.150***	0.018	-1317.903	-0.159***	0.026	-2264.4
Diabetes mellitus	-0.009	0.019	-59.6	-0.099***	0.025	-876.8011	-0.008	0.041	-119.7
Constant	-0.747***	0.032		-0.127**	0.056		0.587***	0.11	
	0.093***	0.016		0.162***	0.026		0.118**	0.046	
	0.649***	0.02		0.923***	0.035		0.896***	0.051	
	1.499***	0.033		1.613***	0.027		1.934***	0.065	
Observations	39,037			13,274			6,046		

Notes: The Extended Estimating Equations (EEE) model estimates were reported in the table; Coef., coefficient; Adjusted Std.err. are standard errors adjusted for clustering at the patient level; UEBMI, Urban Employee-based Basic Medical Insurance scheme; URBMI, Urban Resident-based Basic Medical Insurance scheme; ICU, Intensive Care Unit; PCI, Percutaneous Coronary Intervention; ***, P<0.001, **, P <0.01, *, P <0.05.

Appendix Table 1: Factors associated with direct medical costs and out-of-pocket expenses of ischemic heart disease (Tobit model)

**Associated Factors**	Direct Medical Costs			Out-of-Pocket Expenses		
	n=58,357			n=58,357		
	Coef.	Std.err.	Marginal Effect	Coef.	Std.err.	Marginal Effect
**Gender**						
Female (Reference)						
Male	3,864.085***	198.148	3,003,5	1,447.202***	72.008	1,067.9
**Age group**						
18≤Age<59(Reference)						
60≤Age<69	648.739**	324.225	504.3	-1,167.945***	117.827	-861.8
70≤Age<79	-135.651	299.091	-105.4	-1,781.486***	108.693	-1,314.6
≥80	-1,777.562***	322.882	-1,381.7	-2,606.098***	117.339	-1,923.1
**Insurance type**						
URBMI(Reference)						
UEBMI	726.879**	336.861	565.0	-5,369.495***	122.418	-3,962.2
**Hospital level**						
Primary (Reference)						
Secondary	1,045.374***	342.582	812.5	274.378**	124.496	202.5
Tertiary	11,634.676***	307.273	9,043.4	3,885.602***	111.665	2,867.2
**ICU admission**	112,294.503***	2,610.189	87,284.4	22,045.258***	948.548	16,267.5
**PCI operation**	54,974.505***	271.417	42,730.7	20,313.811***	98.634	14,989.8
**Length of stay(days)**						
Days<15 (Reference)						
15≤Days<30	13,798.356***	237.770	10,725.2	3,040.726***	86.406	2,243.8
Days≥30	41,046.334***	331.025	31,904.6	7,781.409***	120.295	5,742.0
**Year**						
Year 2008 (Reference)						
Year 2009	2,925.639***	353.143	2,274.0	737.715***	128.333	544,4
Year 2010	2,968.288***	355.428	2,307.2	267.011**	129.165	197.0
Year 2011	3,583.690***	329.624	2,785.5	-3,093.429***	119.787	-2,282.7
Year 2012	3,193.745***	318.866	2,482,4	-3,220.872***	115.876	-2,376.7
**Comorbidity**						
None (Reference)						

Hypertension	-2,526.158***	235.770	-1,963.5	-788.663***	85.681	-582.0
Diabetes mellitus	1,242.137***	376.121	965.5	-252.074*	136.687	-186.0

Notes: The Tobit model estimates were reported in the table; Coef., coefficient; Adjusted Std.err. are standard errors adjusted for clustering at the patient level; UEBMI, Urban Employee-based Basic Medical Insurance scheme; URBMI, Urban Resident-based Basic Medical Insurance scheme; ICU, Intensive Care Unit; PCI, Percutaneous Coronary Intervention; ***, *P*<0.001, **, *P* <0.01, *, *P* <0.05.

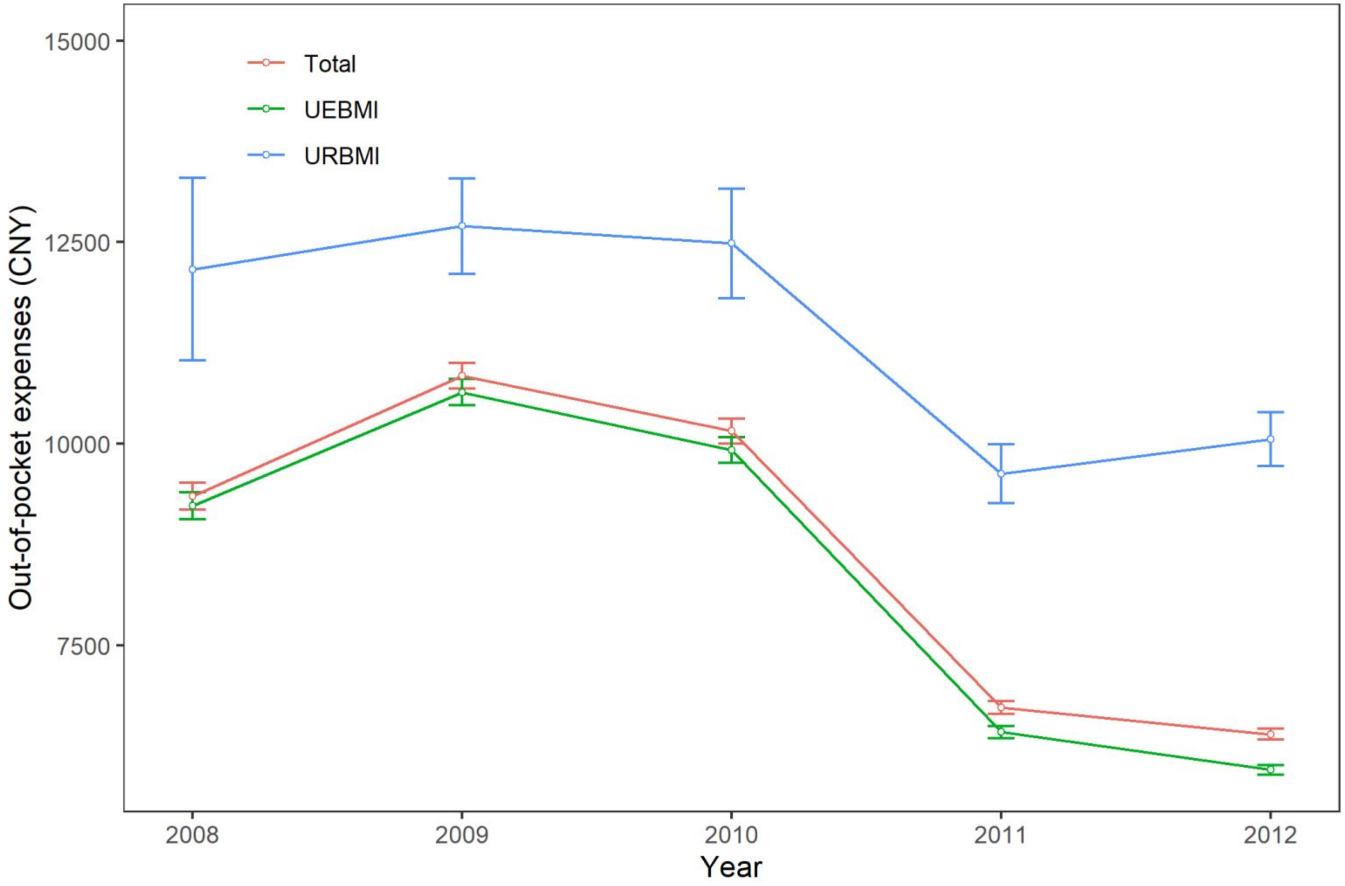

Figure 3 Time trends of out-of-pocket expenses of ischemic heart disease patients.
Notes: All costs were reported on 2012 Chinese Yuan (CNY). UEBMI, Urban Employee-based Basic Medical Insurance; URBMI, Urban Resident-based Basic Medical Insurance.

Reference

Al-Hanawi, M. K., Mwale, M. L., & Qattan, A. M. N. (2021). Health Insurance and Out-Of-Pocket Expenditure on Health and Medicine: Heterogeneities along Income. *Frontiers in Pharmacology,* 12, 638035. doi:10.3389/fphar.2021.638035.

Basu, A., & Rathouz, P. J. (2005). Estimating marginal and incremental effects on health outcomes using flexible link and variance function models. *Biostatistics,* 6(1), 93-109. doi:10.1093/biostatistics/kxh020.

Ding, J. M., Zhang, X. Z., Hu, X. J., Chen, H. L., & Yu, M. (2017). Analysis of hospitalization expenditures and influencing factors for inpatients with coronary heart disease in a tier-3 hospital in Xi'an, China: A retrospective study. *Medicine*, 96(51):e9341. doi: 10.1097/MD.0000000000009341.

Hyun, M. K., & Kan, M. Y. (2022). Association Between Work Status and the Use of Healthcare Services Among Women in the Republic of Korea. *Saf Health Work,* 13(1), 51-58. doi:10.1016/j.shaw.2021.10.004.

Jones, A. (2010). Models for Health Care. (Accessed on 16 March 2023). Available from: https://www.york.ac.uk/media/economics/documents/herc/wp/10_01.pdf

Li, Z., Hung, P., Shi, K., Fu, Y., & Qian, D. (2023). Association of rurality, type of primary caregiver and place of death with end-of-life medical expenditures among the oldest-old population in China. *International Journal for Equity in Health,* 22(1), 1. doi:10.1186/s12939-022-01813-2

Locatelli, C. (2023). Ischemic heart disease. (Accessed on 16 March 2023). Available from: https://www.elder.org/heart-disease-care/ischemic-heart-disease/.

Peng, Z., Zhan, C., Ma, X., Yao, H., Chen, X., Sha, X., & Coyte, P. C. (2021). Did the universal zero-markup drug policy lower healthcare expenditures? Evidence from Changde, China. *Bmc Health Services Research,* 21(1), 1205. doi:10.1186/s12913-021-07211-8

Schlatter, R. P., Hirakata, V. N., & Polanczyk, C. A. (2017). Estimating the direct costs of ischemic heart disease: evidence from a teaching hospital in BRAZIL, a retrospective cohort study. *Bmc Cardiovascular Disorders,* 17(1):180. doi: 10.1186/s12872-017-0615-1.

Tobin, J. (1958). Estimation of Relationships for Limited Dependent Variables. *Econometrica,* 26(1), 24-36.

Walker S, Asaria M, Manca A, Palmer S, Gale CP, Shah AD, et al. (2016). Long-term healthcare use and costs in patients with stable coronary artery disease: a population-based cohort using linked health records (CALIBER). *Eur Heart J Qual Care Clin Outcomes,* 2(2), 125-140. doi:10.1093/ehjqcco/qcw003.

Wang, Q., Zhang, S., Wang, Y. L., Zhang, X. C., & Zhang, Y. (2019). Factors Associated With Hospitalization Costs of Coronary Heart Disease in Township Hospitals in Rural China. *Inquiry-the Journal of Health Care Organization Provision and Financing,* 56:46958019886958. doi: 10.1177/0046958019886958.

Wang, Y., Lei, H., Li, X., Zhou, W., Wang, G., Sun, A., et al.(2022). Lung Cancer-Specific Mortality Risk and Public Health Insurance: A Prospective Cohort Study in Chongqing, Southwest China. *Frontiers in Public Health,* 10, 842844. doi:10.3389/fpubh.2022.842844.

Wei, D., Xiao, W., Zhou, L., Guo, J., Lu, W., & Wang, Y. (2022). Age-Period-Cohort Analysis of Ischemic Heart Disease Morbidity and Mortality in China, 1990-2019. *Circulation journal : official journal of the Japanese Circulation Society,* 86(9), 1437-1443. doi:10.1253/circj.CJ-21-0749.

Yu, S., Zhang, Y., Yao, Y., & Cao, H. (2020). Migraine treatment and healthcare costs: retrospective analysis of the China Health Insurance Research Association (CHIRA) database. *Journal of Headache and Pain,* 21(1), 53. doi:10.1186/s10194-020-01117-2.

Zhang, G., Yu, C., Zhou, M., Wang, L., Zhang, Y., & Luo, L. (2018). Burden of Ischaemic heart disease and attributable risk factors in China from 1990 to 2015: findings from the global burden of disease 2015 study. *BMC Cardiovasc Disord,* 18(1), 18. doi:10.1186/s12872-018-0761-0.

Zhao, Y., Kang, B., Liu, Y., Li, Y., Shi, G., Shen, T.et al. (2014). Health insurance coverage and its impact on medical cost: observations from the floating population in China. *Plos One,* 9(11), e111555. doi:10.1371/journal.pone.0111555.

Zhou, M., Wang, H., Zhu, J., Chen, W., Wang, L., Liu, S., et al. (2016). Cause-specific mortality for 240 causes in China during 1990-2013: a systematic subnational analysis for the Global Burden of Disease Study 2013. *Lancet*, 387(10015), 251-272. doi:Doi 10.1016/S0140-6736(15)00551-6.
